# Resveratrol Attenuates Trimethylamine-*N*-Oxide (TMAO)-Induced Atherosclerosis by Regulating TMAO Synthesis and Bile Acid Metabolism via Remodeling of the Gut Microbiota

**DOI:** 10.1128/mBio.02210-15

**Published:** 2016-04-05

**Authors:** Ming-liang Chen, Long Yi, Yong Zhang, Xi Zhou, Li Ran, Jining Yang, Jun-dong Zhu, Qian-yong Zhang, Man-tian Mi

**Affiliations:** Research Center for Nutrition and Food Safety, Institute of Military Preventive Medicine, Third Military Medical University, Chongqing, People’s Republic of China

## Abstract

The gut microbiota is found to be strongly associated with atherosclerosis (AS). Resveratrol (RSV) is a natural phytoalexin with anti-AS effects; however, its mechanisms of action remain unclear. Therefore, we sought to determine whether the anti-AS effects of RSV were related to changes in the gut microbiota. We found that RSV attenuated trimethylamine-*N*-oxide (TMAO)-induced AS in ApoE^−/−^ mice. Meanwhile, RSV decreased TMAO levels by inhibiting commensal microbial trimethylamine (TMA) production via gut microbiota remodeling in mice. Moreover, RSV increased levels of the genera *Lactobacillus* and *Bifidobacterium*, which increased the bile salt hydrolase activity, thereby enhancing bile acid (BA) deconjugation and fecal excretion in C57BL/6J and ApoE^−/−^ mice. This was associated with a decrease in ileal BA content, repression of the enterohepatic farnesoid X receptor (FXR)-fibroblast growth factor 15 (FGF15) axis, and increased cholesterol 7a-hydroxylase (CYP7A1) expression and hepatic BA neosynthesis. An FXR antagonist had the same effect on FGF15 and CYP7A1 expression as RSV, while an FXR agonist abolished RSV-induced alterations in FGF15 and CYP7A1 expression. In mice treated with antibiotics, RSV neither decreased TMAO levels nor increased hepatic BA synthesis. Additionally, RSV-induced inhibition of TMAO-caused AS was also markedly abolished by antibiotics. In conclusion, RSV attenuated TMAO-induced AS by decreasing TMAO levels and increasing hepatic BA neosynthesis via gut microbiota remodeling, and the BA neosynthesis was partially mediated through the enterohepatic FXR-FGF15 axis.

## IMPORTANCE

Recently, trimethylamine-*N*-oxide (TMAO) has been identified as a novel and independent risk factor for promoting atherosclerosis (AS) partially through inhibiting hepatic bile acid (BA) synthesis. The gut microbiota plays a key role in the pathophysiology of TMAO-induced AS. Resveratrol (RSV) is a natural phytoalexin with prebiotic benefits. A growing body of evidence supports the hypothesis that phenolic phytochemicals with poor bioavailability are possibly acting primarily through remodeling of the gut microbiota. The current study showed that RSV attenuated TMAO-induced AS by decreasing TMAO levels and increasing hepatic BA neosynthesis via gut microbiota remodeling. And RSV-induced hepatic BA neosynthesis was partially mediated through downregulating the enterohepatic farnesoid X receptor-fibroblast growth factor 15 axis. These results offer new insights into the mechanisms responsible for RSV’s anti-AS effects and indicate that the gut microbiota may become an interesting target for pharmacological or dietary interventions to decrease the risk of developing cardiovascular diseases.

## INTRODUCTION

The incidence of cardiovascular diseases (CVDs), such as atherosclerosis (AS), is increasing globally and has become an expensive public health issue (1). A recent metabolomics approach identified that plasma trimethylamine-*N*-oxide (TMAO), a choline metabolite, is a novel and independent risk factor for promoting AS (2, 3). TMAO generation is dependent on the gut microbiota, which first metabolizes dietary choline to trimethylamine (TMA). Thereafter, the TMA is metabolized to TMAO by enzymes of the flavin monooxygenase (FMO) family in the liver (4). Thus, the gut microbiota has been put forward as a key player in the pathogenesis of TMAO-induced AS, which might be a new potential therapeutic target for the prevention and treatment of CVD. It was found that antibiotics (Abs) could attenuate TMAO-caused AS by reducing TMAO synthesis via blocking the pathway of l-carnitine to TMA governed by the gut microbiota (4). However, the side effects and resistance potential of Abs limit their utility. Therefore, the identification of natural products with excellent antimicrobial effects to inhibit gut microbial TMA production might be useful for the prevention and treatment of TMAO-induced AS.

TMAO has been shown to induce AS by affecting cholesterol metabolism through inhibiting hepatic bile acid (BA) synthesis (2–4). Recently, the gut microbiota has been found to play an important role in hepatic BA synthesis (5). Primary BAs are synthesized from cholesterol in the liver and further metabolized by the gut microbiota into secondary BAs via deconjugation, dehydrogenation, and dehydroxylation in the gut (6). This process is controlled by a negative-feedback loop through the activation of the nuclear farnesoid X receptor (FXR) in the ileum and liver (7, 8). Furthermore, many studies have shown that the gut microbiota regulates secondary BA metabolism and BA synthesis in the liver by alleviating the enterohepatic FXR-fibroblast growth factor 15 (FGF15) axis (6, 9). VSL#3 probiotics can induce hepatic BA neosynthesis via downregulation of the gut-liver FXR-FGF15 axis (10). Sayin et al. found that the gut microbiota can reduce the levels of tauro-beta-muricholic acid (T-βMCA), a naturally occurring FXR antagonist. This subsequently inhibits cholesterol 7a-hydroxylase (CYP7A1), the rate-limiting enzyme in BA synthesis, by activating FXR-FGF15 signaling and ultimately regulating BA metabolism in the liver (5, 11). Additionally, tempol, an antioxidant and protective agent against radiation, can alter the gut microbiome, leading to the accumulation of intestinal T-βMCA, thereby inhibiting intestinal FXR signaling and decreasing obesity (12). These results indicate that microbiota-targeted therapies could be effective in preventing gut-related diseases, including AS, by regulating BA metabolism via the enterohepatic FXR-FGF15 axis.

Resveratrol (RSV) is a natural polyphenol that mainly occurs in grapes, berries, and other dietary constituents and is beneficial for treatment of many metabolic diseases, including AS (13). Previous investigations have shown that the physiological effects of dietary RSV are in striking contrast to its poor bioavailability, which is a major concern for the development of this class of compounds into therapeutic agents (14, 15). However, a growing body of evidence supports the hypothesis that phenolic phytochemicals with poor bioavailability are possibly acting primarily through remodeling the gut microbiota. Researchers have confirmed that a polyphenol-rich cranberry extract and metformin attenuate diet-induced metabolic syndrome in mice in a gut microbiota-dependent manner (16, 17). Moreover, it has been found that RSV consumption can significantly modulate the growth of certain gut microbiota *in vivo*, including increasing the *Bacteroidetes*-to-*Firmicutes* ratios, and the growth of *Bacteroides*, *Lactobacillus*, and *Bifidobacterium* (18–22). These results suggested that RSV could be a good candidate for prebiotics and could be used to promote the growth of beneficial commensals and thus to confer health benefits to the host.

Given the close association among TMAO levels, gut microbiota, BA metabolism, and AS, we hypothesized that RSV could attenuate TMAO-induced AS by regulating TMAO synthesis and BA metabolism via the modulation of the gut microbiota. To verify this hypothesis, we examined the effects of RSV on TMAO-induced AS, gut microbiota, TMAO synthesis, and BA metabolism in C57BL/6J and ApoE^−/−^ mice. Furthermore, the potential involvement of the enterohepatic FXR-FGF15 axis was also investigated. We showed, for the first time, that RSV attenuated TMAO-induced AS by decreasing TMAO levels and increasing hepatic BA neosynthesis via gut microbiota remodeling and that RSV-induced BA neosynthesis was partially mediated through the enterohepatic FXR-FGF15 axis.

## RESULTS

### RSV inhibited TMAO synthesis in C57BL/6J mice.

**FIG 1:**
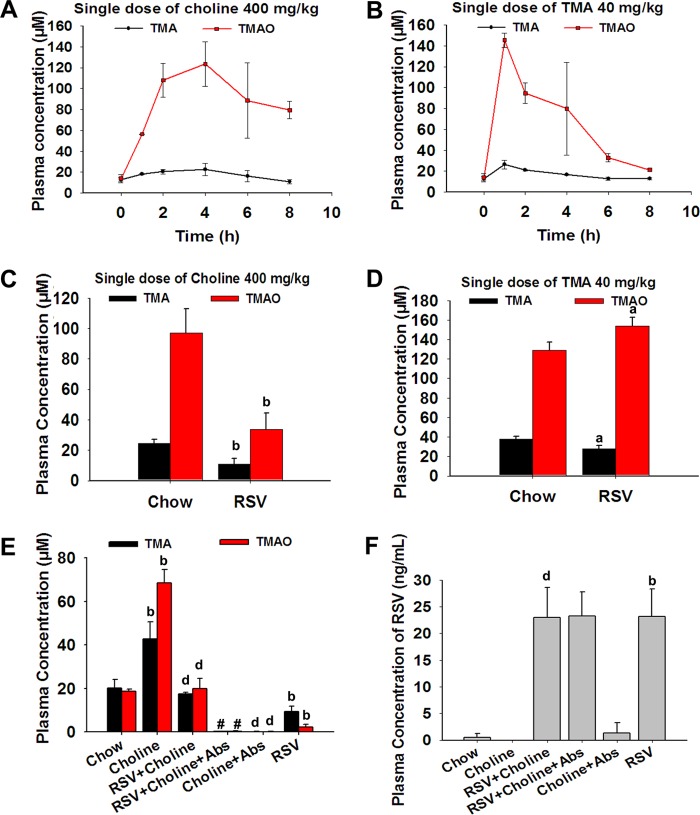
RSV inhibited TMAO synthesis in C57BL/6J mice. (A and B) Eight-week-old female C57BL/6J mice were administered choline (400 mg/kg of body weight, *n* = 10) (A) or TMA (40 mg/kg, *n* = 10) (B). Blood samples were collected at the indicated times. Serum TMA and TMAO levels were determined by LC/MS. (C and D) Eight-week-old female C57BL/6J mice were fed a chow diet with or without RSV (0.4%) in the presence or absence of choline (1%) or Abs. After 30 days, several mice of the chow-and-RSV-fed group were administered choline (400 mg/kg, *n* = 10) (C) or TMA (40 mg/kg, *n* = 10) (D). At 4 h after choline was given, or 1 h after TMA was given, the mice were euthanized and blood was collected. Serum TMA and TMAO levels were determined by LC/MS. (E and F) The other mice were also euthanized, and blood was collected. Serum TMA and TMAO levels (E) and RSV levels (F) were determined by LC/MS. Values are presented as means ± SD (*n* = 10). a, *P* < 0.05; b, *P* < 0.01 (versus vehicle-treated control group); d, *P* < 0.01 (versus choline-treated group); #, *P* < 0.05 (versus group cotreated with choline and RSV).

In order to investigate the effect of RSV on TMAO synthesis in C57BL/6J mice, TMA and TMAO contents in serum were measured at 0, 1, 2, 4, 6 and 8 h after administration of a single dose of choline (400 mg/kg of body weight) or TMA (40 mg/kg) in mice by intragastric gavage. Via liquid chromatography-tandem mass spectrometry (LC/MS), we found that peak concentrations of TMA and TMAO occurred 4 h after choline administration and 1 h after TMA administration (Fig. 1A and B). Thus, in subsequent experiments we used serum samples taken at 4 h or 1 h after the administration of choline or TMA, respectively. Serum TMA and TMAO levels were much lower in RSV-treated mice given a single dose of choline than in those in the control group (Fig. 1C). For RSV-treated mice given TMA, TMAO levels were higher and TMA levels were lower than those in the control group (Fig. 1D), indicating that RSV may induce the metabolism of TMA to TMAO in the liver. Moreover, the addition of 1% choline over 30 days significantly increased the levels of TMA and TMAO in mice; however, this was reversed by RSV administration (Fig. 1E). Additionally, plasma RSV content was higher in mice fed RSV than in mice fed a standard diet (Fig. 1F). Our results suggested that RSV reduced TMAO synthesis levels in C57BL/6J mice.

### RSV inhibited TMAO synthesis by decreasing TMA generation via remodeling microbiota.

**FIG 2:**
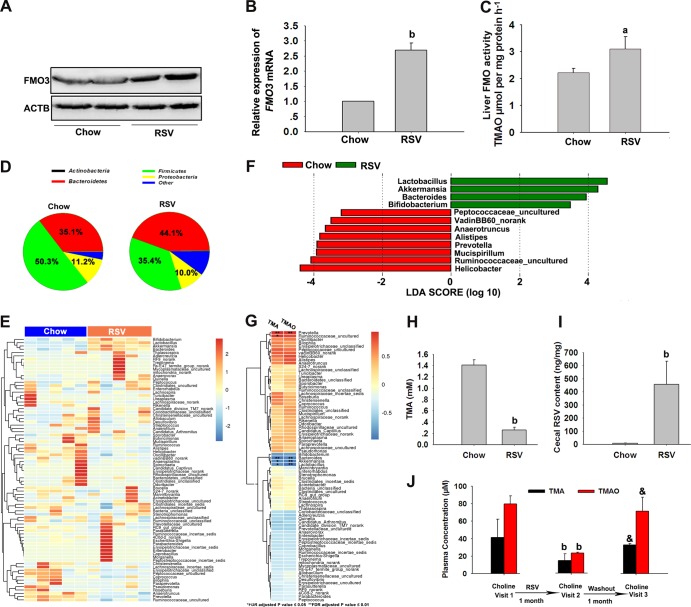
RSV inhibited TMAO synthesis by decreasing TMA generation via remodeling microbiota in C57BL/6J mice. Eight-week-old female C57BL/6J mice (*n* = 5 per group) were fed chow with or without RSV (0.4%) for 30 days. ACTB, β-actin. (A) Western blotting detection of FMO3 in the liver. (B) Expression levels of FMO3 gene mRNAs were quantified using qPCR assays. (C) Liver FMO activity was assessed as described in Materials and Methods. (D) 16S rRNA gene sequencing analysis of cecal content at the phylum level. (E) Heat map of 16S rRNA gene sequencing analysis of cecal content at the genus level. The scale reflects the data as follows: red indicates high values whereas blue indicates low values for the percentages of reads that were classified at that rank. (F) Linear discriminant analysis (LDA) coupled with effect size measurements identifies the taxons most differentially abundant between the chow and RSV diets at the genus level. RSV-diet-enriched taxa are indicated with a positive LDA score (green), and taxa enriched in the normal chow diet have a negative score (red). Only taxa meeting an LDA significant threshold value of >2 are shown. (G) Correlation heat map demonstrating the association between the indicated microbiota taxonomic genera and TMA and TMAO concentrations (all reported as means ± SD in micromoles) from mice grouped by dietary status (chow and RSV). Red denotes a positive association, blue a negative association, and white no association. A single asterisk indicates a significant FDR-adjusted association at *P* values of ≤0.05, and a double asterisk indicates a significant FDR-adjusted association at *P* values of ≤0.01. (H) The production of TMA from choline by cecal content *in vitro.* (I) Cecal RSV content. (J) The same 8-week-old female C57BL/6J mice (*n* = 5 per group) were administered choline (400 mg/kg) at visit 1 (baseline), visit 2 (RSV treatment for 1 month), and visit 3 (washout for 1 month). Four hours after choline was given at each visit, blood samples were obtained from the tail veins. Serum TMA and TMAO levels were determined by LC/MS. Values are presented as means ± SD (*n* = 5). a, *P* < 0.05; b, *P* < 0.01 (versus vehicle-treated control group); &, *P* < 0.01 (versus RSV-treated group).

TMAO generation is dependent on the gut microbiota, which can metabolize dietary choline to TMA. The TMA is then metabolized by enzymes of the FMO family in the liver, among which flavin monooxygenase 3 (FMO3) is the most active (23). Thus, we first examined the effects of RSV on FMO3 expression and activity in the liver. Treatment with RSV resulted in a significant increase in protein and mRNA levels of FMO3 (Fig. 2A and B). RSV also markedly increased FMO activity in the liver (Fig. 2C). These findings corresponded with our earlier results showing that RSV could increase TMAO levels in mice treated with a single dose of TMA (Fig. 1D). These results indicated that the RSV-induced decrease in TMAO levels was not due to its regulation of FMO3 in the liver.

To determine the role of the gut microbiota in the RSV-induced decrease of TMAO levels, we explored bacterial populations in the gut at the phylum and genus levels. Analysis at the phylum level revealed that the bacterial population of vehicle-treated mice was dominated by *Bacteroidetes* (35.1%) and *Firmicutes* (50.3%), with a low level of *Proteobacteria* (11.2%) (Fig. 2D). The proportion of sequences assigned to *Bacteroidetes* was significantly increased in metagenomes of RSV-fed animals at the expense of *Firmicutes* (Fig. 2D)*.* Genus-level analysis showed that RSV induced an increase in the relative abundances of *Bacteroides*, *Lactobacillus*, *Bifidobacterium*, and *Akkermansia* in mice (Fig. 2E and F). RSV administration resulted in a decrease in the relative abundances of *Prevotella*, uncultured *Ruminococcaceae* (*Ruminococcaceae_uncultured*), *Anaerotruncus*, *Alistipes*, *Helicobacter*, and uncultured *Peptococcaceae* (*Peptococcaceae_uncultured*) (Fig. 2E and F). Using Pearson’s correlation tests, we found that, in general, taxa that were present at a relatively high proportion coincident with high plasma TMA concentrations also tended to be present at a relatively high proportion coincident with high plasma TMAO concentrations. Species of several bacterial taxa (*Ruminococcaceae_uncultured*, *Prevotella*, *Bacteroides*, *Akkermansia*, and *Lactobacillus*) remained significantly associated with plasma TMA and/or TMAO levels after false discovery rate (FDR) adjustment for multiple comparisons (Fig. 2G). Via a direct *ex vivo* assay, we found that the production of TMA from choline was lower in cecal content from RSV-treated mice and that the concentration of RSV in the cecal content was much higher than that in the plasma (Fig. 2H and I). Moreover, we found that the same mice fed with RSV could show markedly decreased TMA and TMAO levels after choline treatment; however, after washout with a chow diet for 1 month, they failed to reduce TMA and TMAO contents after choline administration (Fig. 2J). Additionally, real-time quantitative PCR (qPCR) analysis of suspected cecal microbes from mice treated with RSV for 2 months revealed that changes in the gut flora caused by RSV were similar to those observed in mice fed with RSV for 1 month (see Fig. S2A and B in the supplemental material). These results indicated that RSV remodeled the gut microbiome in mice, which might contribute to decreased gut microbial TMA production, thereby inhibiting TMAO synthesis.

### RSV enhanced BA deconjugation and fecal excretion through microbiota modulation.

**FIG 3:**
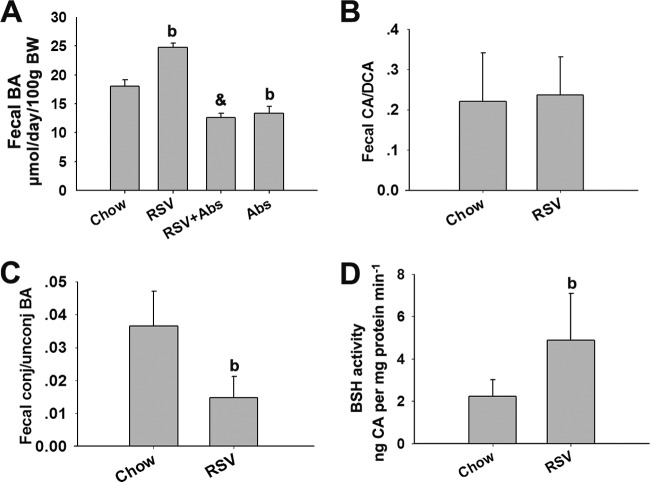
RSV enhanced BA deconjugation and fecal excretion in C57BL/6J mice. Eight-week-old female C57BL/6J mice (*n* = 10 per group) were fed chow with or without RSV (0.4%) in the presence or absence of Abs for 30 days. Fecal samples were collected. (A) Fecal BA excretion was measured by a total BA test (Wako). Fecal BA composition was determined by LC/MS as described in Materials and Methods. (B) CA/DCA ratio. (C) Conjugated/unconjugated (conj/unconj) BA ratio in fecal samples. (D) BSH activity. Values are expressed as means ± SD (*n* = 10). b, *P* < 0.01 (versus vehicle-treated control group); &, *P* < 0.01 (versus RSV-treated group).

The gut microbiota plays an important role in BA metabolism, by mediating deconjugation and 7-α-dehydroxylation of primary BAs (24). To assess the extent of BA biotransformation upon prebiotic colonization, we measured the effects of RSV on fecal BA excretion and composition. RSV significantly enhanced fecal BA loss, which was inhibited by Abs (Fig. 3A). It has been demonstrated that major secondary BAs are deoxycholic acid (DCA) and murideoxy cholic acid (MDCA), which are the 7-α-dehydroxylation products of cholic acid (CA) and beta-muricholic acid (β-MCA), respectively (25). Here, we found that the fecal CA/DCA ratio was unchanged by RSV administration, indicating that RSV had no effect on 7-α-dehydroxylation activity in the gut (Fig. 3B; see also Table S4 in the supplemental material). In contrast, there was a significant decrease in the fecal conjugated/unconjugated BA ratio, along with an increase in bile salt hydrolase (BSH) enzyme activity, in the feces of RSV-treated mice (Fig. 3C and D; see also Table S4). Moreover, we found that RSV treatment resulted in an increase in the proportions of *Lactobacillus* and *Bifidobacterium* that were present in the gut (Fig. 2E and F), likely contributing to its effects on BSH activity. Of note, administration of RSV resulted in a significant reduction in small-intestine (SI) BA content, including taurocholic acid (TCA), TβMCA, CA, chenodeoxycholic acid (CDCA), and DCA, in which the decrease in levels of TCA, CA, CDCA, and DCA might explain nearly 90% of the lower SI BA content (see Fig. S2C and Table S4). It has been demonstrated that the apical sodium bile acid transporter (ASBT) is responsible for ileal BA uptake, with a preference for conjugated over unconjugated BAs (26). We assessed mRNA and protein levels of ASBT and found that RSV had no significant influence on ASBT mRNA or protein levels (see Fig. S2D and E), suggesting that the RSV-mediated increase in fecal BA loss was independent of the presence of ASBT. Finally, we observed a reduction in the mRNA levels of the BA transporters organic solute transporter alpha (OSTα) and beta (OSTβ) in RSV-treated mice, which might contribute to the RSV-induced decrease in ileal BA content (see Fig. S2F). These results suggested that RSV-induced modulation of the gut microbiome contributed to increased BSH activity and subsequently promoted the generation of unconjugated BAs from conjugated BAs, thereby enhancing fecal BA loss.

### RSV induced hepatic BA synthesis in C57BL/6J mice.

**FIG 4:**
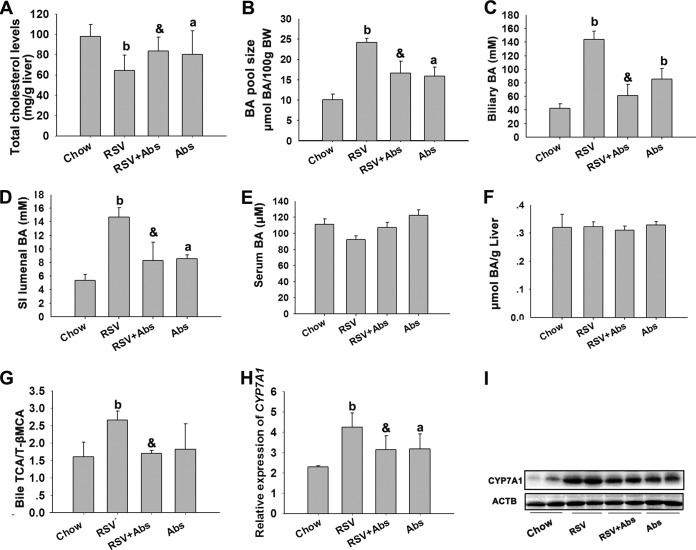
RSV induced hepatic BA synthesis in C57BL/6J mice. Eight-week-old female C57BL/6J mice (*n* = 10 per group) were fed chow with or without RSV (0.4%) in the presence or absence of Abs for 30 days. (A) Liver cholesterol content. (B) The BA pool size was determined for the total BA content of gallbladder bile, liver, and the SI lumenal. (C to F) Total biliary BA content (C) and total BA content in SI lumenal (D), serum (E), and liver (F) were detected. (G) Gallbladder bile samples were subjected to LC/MS, and TCA/TβMCA ratios were calculated. (H) Relative expression levels of the indicated mRNAs in the liver were determined by qPCR. (I) Expression of CYP7A1 was analyzed by Western blotting. Values are expressed as means ± SD (*n* = 10). a, *P* < 0.05; b, *P* < 0.01 (versus vehicle-treated control group); &, *P* < 0.01 (versus RSV-treated group).

It was reported previously that enhanced fecal BA loss is accompanied by enhanced hepatic BA neosynthesis (27, 28). We found that RSV significantly decreased the liver cholesterol level and increased the BA pool size and the gallbladder and SI luminal BA content; these effects were reversed by treatment with Abs (Fig. 4A to D). No differences were observed with respect to serum and hepatic BA content (Fig. 4E and F). In addition, the expression of CYP7A1 mRNA and protein, which is the rate-limiting enzyme in hepatic BA synthesis, was also investigated (29). As shown in Fig. 4G to I and in Table S4 in the supplemental material, RSV increased the bile TCA/TβMCA ratio and induced CYP7A1 mRNA and protein expression and the effects were inhibited by Abs treatment. These results suggested that RSV possibly increased hepatic BA synthesis by inducing CYP7A1 expression in a gut microbiota-dependent manner.

### The enterohepatic FXR-FGF15 axis played a key role in RSV-induced BA synthesis.

**FIG 5:**
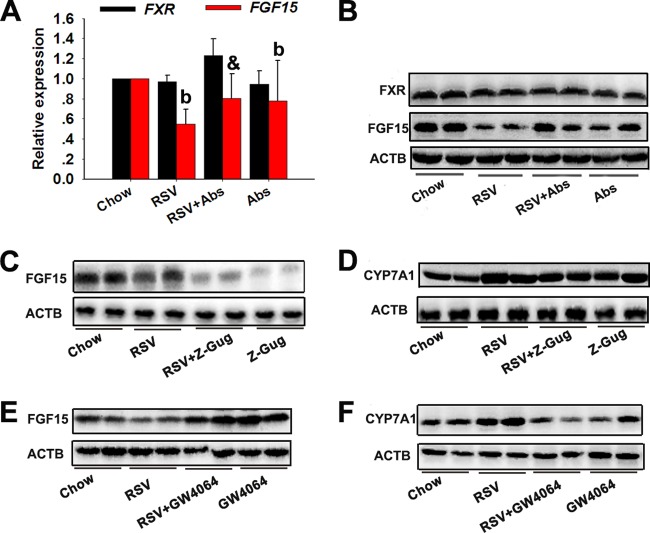
The enterohepatic FXR-FGF15 axis played a key role in RSV-induced BA synthesis. Eight-week-old female C57BL/6J mice (*n* = 10 per group) were fed chow with or without RSV (0.4%) for 30 days. Z-Gug (100 mg/kg body weight) or GW4064 (75 mg/kg body weight) was given 7 days prior to surgical procedures. (A) Relative expression levels of ileal FXR and FGF15 gene mRNAs. (B) Western blotting was used to detect ileal FXR and FGF15 expression. (C) Expression of the indicated proteins in ileal tissues. (D) Liver CYP7A1 expression was detected by Western blotting. (E) FGF15 expression in ileal tissues. (F) CYP7A1 expression in liver samples. Values are expressed as means ± SD (*n* = 10). b, *P* < 0.01 (versus vehicle-treated control group); &, *P* < 0.01 (versus RSV-treated group).

The feedback suppression of CYP7A1 gene transcription by FXR is one of the most important mechanisms in the maintenance of BA homeostasis (30). Previous studies have shown that FXR activation is a major mechanism in the suppression of BA synthesis and induces small heterodimer partner (SHP) and FGF15, which work together to suppress CYP7A1 (31, 32). Thus, we sought to determine the possible roles of the gut-liver FXR signaling in RSV-induced BA synthesis. RSV treatment resulted in a significant decrease in ileal FGF15 mRNA and protein levels; however, ileal FXR mRNA and protein levels were unchanged (Fig. 5A and B). These observations suggested that RSV downregulated FXR transcriptional activity, as demonstrated by the reduction in mRNA levels of its target genes. In the liver, RSV treatment appeared to have no significant effect on the mRNA level of the SHP gene and the mRNA and protein levels of FXR (see Fig. S3A and B in the supplemental material). These findings highlighted that RSV downregulated the ileal FXR-FGF15 axis, which might result from RSV-induced significant reduction in FXR agonists (TCA, DCA, CA, and CDCA) in SI tissues (see Table S4). Furthermore, we used Z-Guggulsterone (Z-Gug), a natural antagonist of FXR, to suppress FXR in mice as outlined previously (33). Treatment with Z-Gug markedly inhibited the expression of FGF15, thereby inducing CYP7A1 expression in mice (Fig. 5C and D). Additionally, similar results were seen in mice cotreated with RSV and Z-Gug (Fig. 5C and D). The presence of synthetic FXR agonist GW4064 was able to reverse RSV-induced alterations in the expression of FGF15 and CYP7A1 in mice (Fig. 5E and F). Taken together, these observations indicated that the gut-liver FXR-FGF15 axis played a key role in RSV-induced BA synthesis.

### RSV protected ApoE^−/−^ mice from TMAO-induced AS in a gut microbiota-dependent manner.

**FIG 6:**
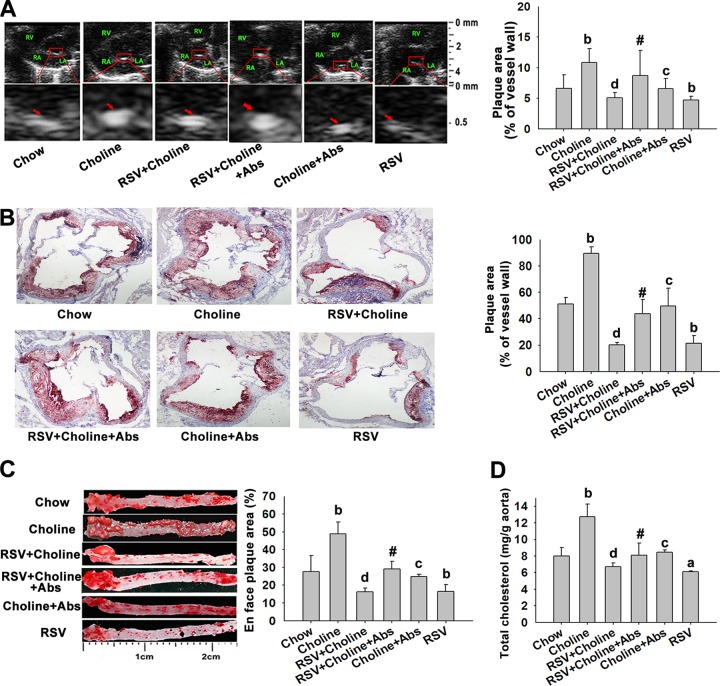
RSV protected ApoE^−/−^ mice from TMAO-induced AS. Eight-week-old female ApoE^−/−^ mice (*n* = 10 per group) were given RSV (0.4%) with or without choline (1%) in the absence or presence of Abs for 4 months. The control group was fed with a chow diet. (A) Ultrasound B-mode images of the aortic sinus and quantification. Arrows indicate the regions of interest. RV, right ventricle; RA, right atrium; LA, left atrium. (B) Oil red O-stained aortic roots (counterstained with hematoxylin) and quantification. (C) Oil red O staining of whole aortas, including the aortic arch, thoracic, and abdominal regions, and their quantitation. (D) Total cholesterol content in the thoracic and abdominal aorta. Values are expressed as means ± SD (*n* = 10). a, *P* < 0.05; b, *P* < 0.01 (versus vehicle-treated control group); c, *P* < 0.05; d, *P* < 0.01 (versus choline-treated group); #, *P* < 0.05 (versus group cotreated with RSV and choline).

Plasma TMAO is strongly associated with AS (2). Therefore, we investigated whether changes in TMAO induced by RSV through microbiota remodeling were associated with AS regression in ApoE^−/−^ mice. The atherosclerotic lesion area and the cholesterol content in the whole aorta, as indexes of AS severity, were much higher in mice fed with choline than in chow-fed mice, and the effects were markedly reversed by RSV or Abs administration (Fig. 6). Moreover, when the gut microbiota was suppressed by Abs, RSV-induced inhibition of TMAO-caused AS was significantly abolished (Fig. 6). Additionally, RSV single treatment could also attenuate the pathogenesis of AS in ApoE^−/−^ mice (Fig. 6). These results suggested that RSV attenuated TMAO-induced AS in a gut microbiota-dependent manner.

### RSV reduced TMAO levels by decreasing TMA generation via remodeling microbiota in ApoE^−/−^ mice.

**FIG 7:**
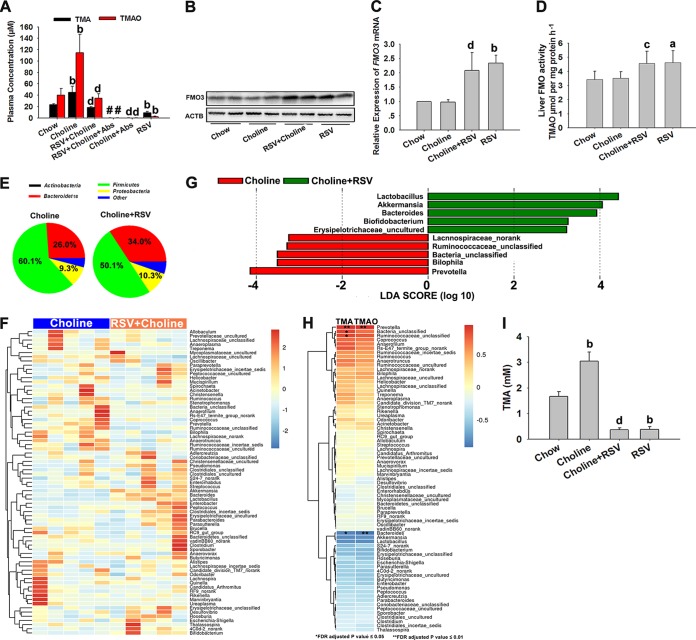
RSV reduced TMAO levels by decreasing TMA generation via remodeling microbiota in ApoE^−/−^ mice. Eight-week-old female ApoE^−/−^ mice (*n* = 5 per group) were fed chow, chow with RSV (0.4%), chow with choline (1%), or chow with choline (1%) plus RSV (0.4%) in the absence or presence of Abs for 4 months. (A) Serum TMA and TMAO levels were measured by LC/MS. (B) Western blotting detection of FMO3 expression in the liver. (C) Expression levels of FMO3 gene mRNAs were quantified using qPCR assays. (D) Liver FMO activity was assessed as described in Materials and Methods. (E) 16S rRNA gene sequencing analysis of cecal content at the phylum level. (F) Heat map of 16S rRNA gene sequencing analysis of cecal content at the genus level. The scale reflects the data as follows: red indicates high values whereas blue indicates low values for the spercentage of reads that were classified at that rank. (G) Linear discriminant analysis (LDA) coupled with effect size measurements identifies the most differentially abundant taxons at the genus level between the choline diet and the RSV-plus-choline diet. RSV-plus-choline-diet-enriched taxa are indicated with a positive LDA score (green), and taxa enriched in the choline diet have a negative score (red). Only taxa meeting an LDA significance threshold of >2 are shown. (H) Correlation heat map demonstrating the association between the indicated microbiota taxonomic genera and TMA and TMAO concentrations (all reported as means ± SD in micromoles) from mice grouped by dietary status (choline and RSV plus choline). Red denotes a positive association, blue a negative association, and white no association. A single asterisk indicates a significant FDR-adjusted association of *P* values of ≤0.05, and a double asterisk indicates a significant FDR-adjusted association of *P* values of ≤0.01. (I) Production of TMA from choline by cecal content *in vitro*. Values are expressed as means ± SD (*n* = 5). a, *P* < 0.05; b, *P* < 0.01 (versus vehicle-treated control group); c, *P* < 0.05; d, *P* < 0.01 (versus choline-treated group); #, *P* < 0.05 (versus group cotreated with RSV and choline).

As shown in Fig. 7A to D, RSV administration markedly decreased TMA and TMAO contents and increased the expression levels of liver FMO3 and FMO activity. These results were consistent with our findings in C57BL/6J mice, suggesting the important role of the gut microbiota in the RSV-induced decrease of TMAO levels.

The composition of the gut microbiota in cecal content was then determined. Via qPCR and sequencing 16S rRNA assays, we found that choline administration markedly increased the bacterial concentrations of *Bacteroidetes* phyla and decreased the concentrations of *Firmicutes* phyla compared to the control group (see Fig. S4A in the supplemental material). The *Proteobacteria* and *Actinobacteria* phyla showed no significant changes after choline treatment in ApoE^−/−^ mice (see Fig. S4A). Moreover, compared to chow-fed ApoE^−/−^ mice, choline significantly increased the number of *Prevotella* and decreased the concentrations of *Bacteroides*, *Lactobacillus*, and *Bifidobacterium* (see Fig. S4B)*.* These results indicated that chronic choline supplementation might alter cecal microbial composition, thereby enhancing the synthesis of TMA and TMAO and ultimately increasing AS. As expected, treatment with RSV resulted in an increase in *Bacteroidetes* abundance (from 20.6 to 34.0%) at the expense of *Firmicutes* (from 60.1 to 50.1%) in choline-treated ApoE^−/−^ mice (Fig. 7E). RSV also increased the relative abundances of *Bacteroides*, *Lactobacillus*, *Bifidobacterium*, and *Akkermansia* and decreased the relative abundances of *Prevotella*, *Ruminococcaceae_unclassified*, and *Biophila* in choline-fed ApoE^−/−^ mice (Fig. 7F and G). After FDR adjustment for multiple comparisons, the abundance of genera *Prevotella* and *Bacteroides* was still significantly associated with TMAO and TMA levels in choline-treated ApoE^−/−^ mice (Fig. 7H). Additionally, via a direct *ex vivo* assay, we found that the production of TMA from choline was higher in cecal content from choline-treated mice and that the effect was reversed by RSV administration (Fig. 7I). Combined with the results seen in C57BL/6J mice, we postulated that RSV attenuated TMAO-induced AS by reducing TMAO synthesis via inhibition of the gut microbial TMA production.

### RSV induced hepatic BA synthesis in choline-treated ApoE^−/−^ mice.

**FIG 8:**
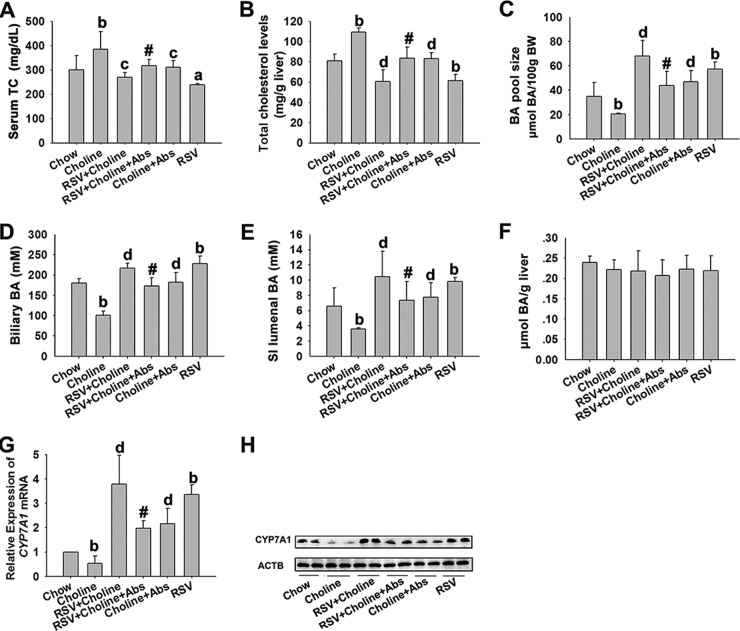
RSV induced hepatic BA synthesis in choline-treated ApoE^−/−^ mice. Eight-week-old female ApoE^−/−^ mice (*n* = 10 per group) were fed chow, chow with RSV (0.4%), chow with choline (1%), or chow with choline (1%) plus RSV (0.4%) in the absence or presence of Abs for 4 months. (A) Serum TC levels were measured enzymatically using commercially available kits. (B) Liver cholesterol content. (C) The BA pool size was determined for the total BA content of gallbladder bile, liver, and the SI lumenal. BW, body weight. (D to F) Total biliary BA content (D) and total BA content in SI lumenal (E) and liver (F) were detected. (G) Relative expression levels of the indicated mRNAs in the liver were determined by qPCR. (H) Expression of CYP7A1 was analyzed by Western blotting. Values are expressed as means ± SD (*n* = 10). a, *P* < 0.05; b, *P* < 0.01 (versus vehicle-treated control group); c, *P* < 0.05; d, *P* < 0.01 (versus choline-treated group); #, *P* < 0.05 (versus choline and RSV-cotreated group).

TMAO appears to contribute to the development of AS, in part by affecting cholesterol metabolism through the regulation of the BA synthesis pathway (4). Therefore, the influence of RSV on BA synthesis was investigated in choline-treated ApoE^−/−^ mice. We found that the presence of choline significantly increased serum total cholesterol (TC) and liver cholesterol levels and decreased BA pool size and gallbladder and SI luminal BA content in ApoE^−/−^ mice; these effects were reversed by treatment with RSV (Fig. 8A to E). And there were no significant differences in liver BA content among the groups (Fig. 8F). Meanwhile, RSV also increased the bile TCA/TβMCA ratio (see Fig. S5A and Table S4 in the supplemental material) and reversed the choline-induced decrease of CYP7A1 mRNA and protein expression in ApoE^−/−^ mice (Fig. 8G and H). However, when the gut microbiota was suppressed by Abs, RSV-induced hepatic BA synthesis was significantly inhibited in choline-fed ApoE^−/−^ mice (Fig. 8). Additionally, RSV single treatment could also induce hepatic BA synthesis in ApoE^−/−^ mice (Fig. 8). These results suggested that RSV induced hepatic BA synthesis in a gut microbiota-dependent manner in choline-treated ApoE^−/−^ mice.

### RSV downregulated the enterohepatic FXR-FGF15 axis in choline-treated ApoE^−/−^ mice.

**FIG 9:**
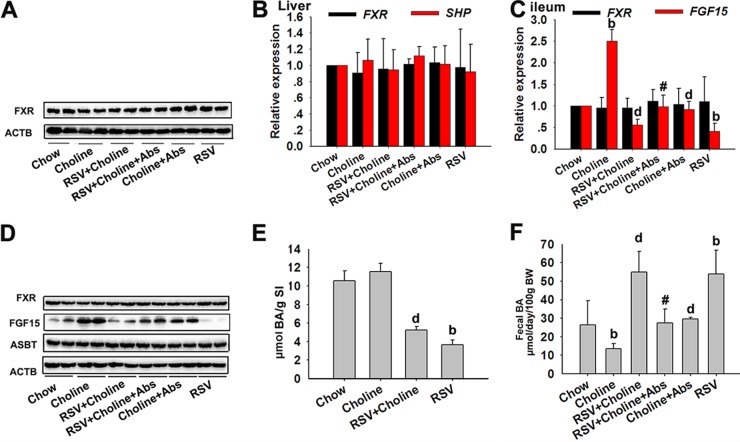
RSV downregulated the enterohepatic FXR-FGF15 axis in choline-treated ApoE^−/−^ mice. Eight-week-old female ApoE^−/−^ mice (*n* = 10 per group) were fed chow, chow with RSV (0.4%), chow with choline (1%), or chow with choline (1%) plus RSV (0.4%) in the absence or presence of Abs for 4 months. (A) Expression of CYP7A1 was analyzed by Western blotting. (B and C) Relative expression levels of the indicated mRNAs in the liver (B) and ileal tissue (C) were determined by qPCR. (D) Western blotting was used to detect ileal FXR and FGF15 expression. (E and F) BA content in SI tissue (E) and fecal samples (F). b, *P* < 0.01 (versus vehicle-treated control group); d, *P* < 0.01 (versus choline-treated group); #, *P* < 0.05 (versus group cotreated with choline and RSV).

Finally, the effects of RSV on gut-liver FXR signaling were determined in choline-treated ApoE^−/−^ mice. The mRNA level of the SHP gene and the mRNA and protein levels of FXR in the liver were unchanged by RSV (Fig. 9A and B). The ileal FGF15 mRNA and protein levels were markedly reduced by RSV; however, ileal FXR mRNA and protein levels were unchanged in choline-fed ApoE^−/−^ mice (Fig. 9C and D). In addition, RSV had no effect on ASBT expression but still decreased the SI BA content, including TCA, CDCA, and TβMCA, in choline-fed ApoE^−/−^ mice (Fig. 9D and E; see also Table S4 in the supplemental material). RSV also increased fecal BA loss and fecal conjugated/unconjugated BA levels and increased BSH activity, with fecal CA/DCA ratios unchanged in choline-fed ApoE^−/−^ mice (Fig. 9F; see also Fig. S5B to D and Table S4). These results were similar to those found in C57BL/6J mice. Overall, we came to the conclusion that RSV attenuated TMAO-induced AS, partially by regulating BA synthesis through the enterohepatic FXR-FGF15 axis in a gut microbiota-dependent manner.

## DISCUSSION

In the current study, the role of the gut microbiota in the protective effects of RSV against AS was determined in mice. We found that RSV reduced TMAO levels by inhibiting gut microbial TMA formation via remodeling gut microbiota, thereby attenuating TMAO-induced AS. To the best of our knowledge, we are the first to demonstrate the role of the gut microbiota in RSV-induced protection against AS. Dietary choline is metabolized by the intestinal microbiota to TMA, which is further metabolized by FMO enzymes, in particular, FMO3, to produce TMAO in the liver (23). Recently, plasma TMAO was identified as a metabolite strongly associated with AS (2–4). Researchers confirmed that feeding AS-prone mice diets enriched in either choline or TMAO enhanced the development of AS. Enhanced AS, as observed by dietary choline supplementation, is entirely dependent on the gut microbiota. Treatment with Abs, or germ-free conditions, abolished dietary choline-driven TMAO generation and the development of AS (2, 4). We observed that RSV attenuated TMAO-induced AS and reduced TMA and TMAO levels in mice. Moreover, RSV increased the expression and activity of FMO3 in the liver, corresponding with previously published results (34). It has been demonstrated that the gene encoding FMO3 is a direct FXR target gene, and FXR has been found to be a target of sirtuin 1 (SIRT1) in metabolic regulation (23, 34, 35). Moreover, RSV is a well-known activator of SIRT1 and our previous study also found that RSV improved hepatic steatosis partially by inducing autophagy via the SIRT1 signaling pathway *in vitro* and *in vivo* (36–38). Therefore, RSV might increase liver FMO3 expression and activity via regulation of the SIRT1-FXR signaling pathway. However, the exact underlying mechanisms need to be further elucidated. Our findings suggested that RSV-induced TMAO reduction was not due to its regulation of FMO3 in the liver.

Previous results suggested possible prebiotic benefits associated with the inclusion of red wine polyphenols, especially RSV, in a diet (20). Qiao et al. (21) demonstrated that RSV improves the gut microbiota dysbiosis induced by a high-fat diet, including increasing the *Bacteroidetes*-to-*Firmicutes* ratios, significantly inhibiting the growth of *Enterococcus faecalis*, and increasing the growth of *Lactobacillus* and *Bifidobacterium* in mice. However, Etxeberria et al. (22) claimed that RSV supplementation alone or in combination with quercetin supplementation scarcely modified the profile of gut bacteria but acted at the intestinal level, altering the mRNA expression of tight-junction proteins and inflammation-associated genes in high-fat sucrose diet-fed rats. Here, we found that RSV inhibited TMAO synthesis by decreasing gut microbial TMA production through gut microbiota modulation, which subsequently attenuated TMAO-induced AS. Meanwhile, a recent report showed that 3,3-dimethyl-1-butanol can reduce TMAO levels by inhibiting the formation of TMA from microbes in mice, thereby attenuating choline diet-enhanced AS (39). These results indicated that targeting gut microbial production of TMA specifically and of nonlethal microbial inhibitors in general may serve as a potential therapeutic approach for the treatment of CVD. In addition, the physiological effects of dietary RSV appeared to be in striking contrast to its poor bioavailability, which has been a major concern in the development of this class of compounds into therapeutic agents (14, 15). Our findings suggested that RSV likely exerted its primary effects by remodeling gut microbiota. This might explain the paradox that RSV has low bioavailability in humans while exerting noticeable bioactivities. These findings provide a new insight into the potential mechanisms responsible for the cardiovascular protective effects of RSV and indicate that the gut microbiota may play an important role.

Furthermore, it has been found that Abs could attenuate TMAO-caused AS by reducing TMAO synthesis via blocking the pathway of choline to TMA governed by the gut microbiota (4). Our results indicated that Abs treatment could markedly inhibit the RSV-induced decrease of AS in choline-fed ApoE^−/−^ mice, which suggests that RSV’s protective effects on the proatherogenic phenotype might depend on other factors of the microbiota beside reducing gut microbial TMA production. Current evidence suggests that TMAO appears to contribute to the development of AS, in part by regulating cholesterol elimination and BA synthesis (2, 4). BAs are synthesized from cholesterol by CYP7A1 in the liver and play a central role in cholesterol homoeostasis (8). The gut microbiota is important for BA metabolism, as it mediates primary BA deconjugation and subsequent conversion to secondary BAs, which are responsible for BA synthesis in the liver (5). The gut microbiome produces potent ligands corresponding to BA receptors; probiotics or prebiotics could act as therapeutics of BA dysmetabolism, indicating that microbiota-targeted therapies could be effective in preventing and/or treating gut-related diseases, including AS (40). We observed that RSV significantly increased the expression of CYP7A1, subsequently inducing BA synthesis in the liver. RSV also increased the proportions of *Lactobacillus* and *Bifidobacterium*, which are considered BSH-active bacteria (41). Bacterial BSH activity affects systemic metabolic processes and adiposity in the host. It also represents a key mechanistic target for the control of obesity and hypercholesterolemia, acting by deconjugating BAs to generate unconjugated BAs (42). For the first time, we found that RSV induced hepatic BA synthesis by remodeling the gut microbiome. Dietary RSV has been shown to increase the expression of hepatic CYP7A1 and to ameliorate hypercholesterolemia in high-fat-fed C57BL/6J mice (43). Our findings were the first to indicate the crucial role of the gut microbiota in RSV-induced cholesterol metabolism in the liver, indicating that RSV could attenuate TMAO-caused AS, partially by inducing BA synthesis in a gut microbiota-dependent manner. These findings offer new insights into the protective effects of RSV against AS.

We also investigated the potential involvement of ASBT and the enterohepatic FXR-FGF15 axis in RSV-mediated BA synthesis. Recently, it was found that ASBT plays a key role in the enterohepatic recycling of BAs and indirectly contributes to cholesterol homoeostasis (26). RSV promotes the degradation of ASBT *in vitro*, which might have some clinical relevance with regard to the observed cholesterol-lowering effects of RSV (44). We found that RSV reduced ileal BA levels, with no effect on ASBT expression. Our results contradict those previously published, possibly because we were experimenting *in vivo*, which is a more complex environment than that *in vitro*. Moreover, we observed a reduction in the mRNA levels of the BA transporters encoded by the OSTα and OSTβ genes in RSV-treated mice; that reduction might contribute to the RSV-induced decrease in ileal BA content. Meanwhile, we found that RSV increased BSH activity, thereby enhancing BA deconjugation. This might also result in the decrease in ileal BA content, because the ileal BA transporters prefer conjugated BAs to unconjugated BAs (26). Interestingly, the SI lumenal BA content was higher upon RSV treatment, which was presumably a result of increased biliary output. However, the exact underlying mechanisms need to be further clarified. It has been reported that enhanced fecal BA loss, either driven by ASBT genetic deletion or induced by BA sequestrant administration, is accompanied by enhanced hepatic BA neosynthesis (27, 28). We showed that RSV induced BA synthesis in the liver, contributing to its protective effect on AS caused by TMAO. These findings provide new insights into the cardiovascular benefits of RSV.

FXR is highly expressed in the liver and intestine, where it functions as an intracellular sensor of BAs. FXR is required for the negative-feedback regulation of BA biosynthesis and the enterohepatic cycle (30). Previous studies have shown that FXR activation induces SHP and FGF15, thereby suppressing CYP7A1 expression and ultimately inhibiting BA synthesis (31, 32). BA synthesis and modulation of the enterohepatic FXR-FGF15 axis are seen in germ-free, gnotobiotic, and antibiotic-treated animals (5). Results from a recent study demonstrate that probiotic VSL#3 affects hepatic BA synthesis by downregulating the gut-liver FXR-FGF15 axis. This suggests that microbiota-targeted therapies affect cholesterol metabolism by inducing hepatic BA synthesis via the regulation of the gut-liver FXR-FGF15 axis (10). We also found that RSV decreased the activity of ileal FXR and the expression of FGF15 by decreasing the levels of FXR agonists (TCA and CDCA) in SI tissues (45, 46). When Z-Gug, a natural antagonist of FXR, was administered, RSV failed to reduce FGF15 expression and to induce the expression of CYP7A1 in mice. Additionally, the presence of GW4064, a synthetic FXR agonist, reversed RSV-induced alterations in FGF15 and CYP7A1 expression. These observations indicated that the gut-liver FXR-FGF15 axis was required for RSV to induce hepatic BA synthesis.

Finally, it is not known whether TMAO interacts directly with a specific receptor or whether it acts to alter signaling pathways indirectly by altering the protein conformation (that is, via allosteric effects), whereas TMA has been reported to influence signal transduction by direct interaction with a family of G protein-coupled receptors (47). TMAO, a small quaternary amine with an aliphatic character, is reportedly capable of directly inducing conformational changes in proteins and of stabilizing protein folding and acting as a small-molecule protein chaperone (48, 49). It is thus conceivable that TMAO may alter many signaling pathways without directly acting at a “TMAO receptor.” However, the exact mechanisms by which circulating TMAO promotes AS and whether RSV could also reverse TMAO-caused changes in other signaling pathways are still largely unknown and need to be further elucidated.

**FIG 10:**
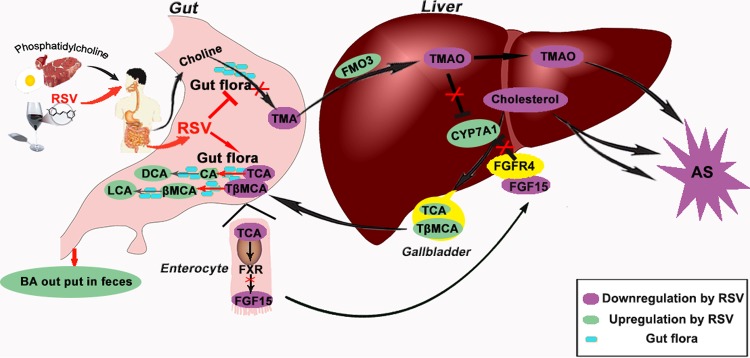
A model depicting use of RSV to attenuate AS by targeting gut microbiota. Dietary RSV can alter the composition of gut flora. On the one hand, this can result in reduced levels of gut microbial TMA production, subsequently leading to decreased TMAO synthesis in the liver and, ultimately, to inhibition of AS. On the other hand, RSV increases BSH activity by gut microbiota remodeling, which promotes the generation of unconjugated BAs from conjugated BAs and enhances fecal BA loss. RSV-induced fecal BA loss leads to a decrease in ileal BA content, thereby inhibiting the ileal FXR-FGF15 axis, and then increases the expression levels of CYP7A1 in the liver, subsequently inducing hepatic BA neosynthesis which contributes to cholesterol homeostasis, finally attenuating AS. Red indicates the effect of RSV, purple indicates downregulation by RSV, green indicates upregulation by RSV, and blue indicates the gut flora. LCA, lithocholic acid.

To the best of our knowledge, we have demonstrated for the first time that RSV attenuated TMAO-induced AS by regulating the synthesis of TMAO and BAs via remodeling of the gut microbiota and that RSV-mediated hepatic BA neosynthesis was partially modulated by downregulation of the gut-liver FXR-FGF15 axis (Fig. 10). Hence, these results open a new avenue of research regarding the potential cardiovascular protective effects of RSV and indicate that the gut microbiota may become an interesting target for pharmacological or dietary interventions to decrease the risk of developing CVD.

## MATERIALS AND METHODS

### Animals and treatments.

Female C57BL/6J mice and ApoE^−/−^ mice with a C57BL/6 genetic background were purchased from Jackson Laboratory (Bar Harbor, ME, USA). Animals were maintained at a controlled temperature (22 ± 2°C), with a 12-h light/dark period. For all the experiments, mice were housed individually in cages and had *ad libitum* access to water. Mice of the control group were fed a standard chow diet (NIH31 modiﬁed mouse/rat diet; Harlan Teklad). Animal experiments were carried out in strict accordance with the recommendations in the *Guide for the Care and Use of Laboratory Animals* published by the National Institutes of Health and were approved by the Animal Care and Use Committee of the Third Military Medical University (Chongqing, China; Approval SYXC-2014-00112). Pentobarbital sodium anesthesia (50 mg/kg of body weight) was administered prior to surgical procedures, with maximal effort exerted to minimize suffering. At the end of the experiments, mice were killed by cervical dislocation followed by decapitation.

### Statistical analyses.

Quantitative data are presented as means ± standard deviations (SD) of results from three independent experiments. Statistical analysis was conducted with either Student’s *t* test or one-way analysis of variance using SPSS 13.0 (SPSS Inc., Chicago, IL). A *P* value less than 0.05 was considered statistically significant, and the Tukey-Kramer *post hoc* test was applied if the *P* value was less than 0.05. Correlations between host parameters and bacterial populations were assessed by Pearson’s correlation tests using GraphPad Prism version 6.00 (GraphPad Software, La Jolla, CA). FDR in the multiple comparisons were estimated for each taxon based on the *P* values that resulted from correlation estimates in R statistical software.

Full descriptions of additional materials and methods are given in Text S1 in the supplemental material.

## SUPPLEMENTAL MATERIAL

Figure S1  Experimental design. The schematic depicts the overall design of the animal experiment to evaluate the effect of RSV on TMAO-induced AS in both C57BL/6J (A) and ApoE^−/−^ (B) mice. Download Figure S1, TIF file, 1.7 MB

Figure S2  RSV remodeled gut microbiota and decreased ileal BA content without modifying ASBT function. Eight-week-old female C57BL/6J mice (*n* = 10 per group) were fed a chow diet with or without RSV (0.4%) for 1 or 2 months. (A and B) The relative abundances of the indicated bacterial strains at the phylum level (A) and the genus level (B) in the cecal content were assessed by qPCR assay. (C) Total BA content in ileal tissues. (D) Western blotting was used to detect the expression of ASBT. (E and F) Expression of ASBT gene mRNA (E) and OSTα and OSTβ gene mRNA (F) was determined by qPCR assays. Values are expressed as means ± SD (*n* = 10). a, *P* < 0.05; b, *P* < 0.01 (versus vehicle-treated control group). Download Figure S2, TIF file, 0.2 MB

Figure S3  RSV had no effect on liver FXR and SHP gene mRNA expression. Eight-week-old female C57BL/6J mice (*n* = 10 per group) were fed a chow diet with or without RSV (0.4%) in the presence or absence of Abs for 30 days. Liver tissues were collected. (A) Relative expression levels of the indicated mRNAs in the liver were determined by qPCR assays. (B) FXR expression was analyzed by Western blotting. Values are expressed as means ± SD (*n* = 10). Download Figure S3, TIF file, 1.9 MB

Figure S4  RSV remodeled gut microbiota in choline-fed ApoE^−/−^ mice. Eight-week-old female ApoE^−/−^ mice (*n* = 10 per group) were fed chow, chow with RSV (0.4%), chow with choline (1%), or chow with choline (1%) plus RSV (0.4%) for 4 months. The relative abundances of the indicated bacterial strains at the phylum level (A) and the genus level (B) in the cecal content were assessed by qPCR assay. a, *P* < 0.05; b, *P* < 0.01 (versus vehicle-treated control group); d, *P* < 0.01 (versus choline-treated group). Download Figure S4, TIF file, 0.1 MB

Figure S5  RSV altered the BA composition in gallbladder bile and feces and increased fecal BSH activity in choline-treated ApoE^−/−^ mice. Eight-week-old female ApoE^−/−^ mice (*n* = 10 per group) were fed chow, chow with choline (1%), or chow with choline (1%) plus RSV (0.4%) for 4 months. The BA composition of gallbladder bile and feces was analyzed by LC/MS. (A) TCA/TβMCA ratios in gallbladder bile. (B) Conjugated/unconjugated BA ratios in fecal samples. (C) Fecal BSH activity. (D) CA/DCA ratios in fecal samples. Values are expressed as means ± SD (*n* = 10). a, *P* < 0.05; b, *P* < 0.01 (versus vehicle-treated control group); d, *P* < 0.05 (versus choline-treated group). Download Figure S5, TIF file, 0.5 MB

Table S1  Primers used for qPCR. Table S1, DOCX file, 0.02 MB

Table S2  Composition of Mega medium. Table S2, DOCX file, 0.02 MB

Table S3  Retention times of BAs. Table S3, DOCX file, 0.02 MB

Table S4  Biliary, SI and fecal bile acid composition profile in mice (related to data shown in Fig. 3, 4, and 9 and in Fig. S2 and S5). Table S4, DOCX file, 0.02 MB

Text S1  Supplemental materials and methods. Download Text S1, DOCX file, 0.1 MB
