# Supplementary figures and images for: Resveratrol Attenuates Trimethylamine-N-Oxide (TMAO)-Induced Atherosclerosis by Regulating TMAO Synthesis and Bile Acid Metabolism via Remodeling of the Gut Microbiota
Source: mBio. 2016 Apr 5;7(2):e02210-15. doi: 10.1128/mBio.02210-15 (PMC4817264; doi:10.1128/mBio.02210-15)

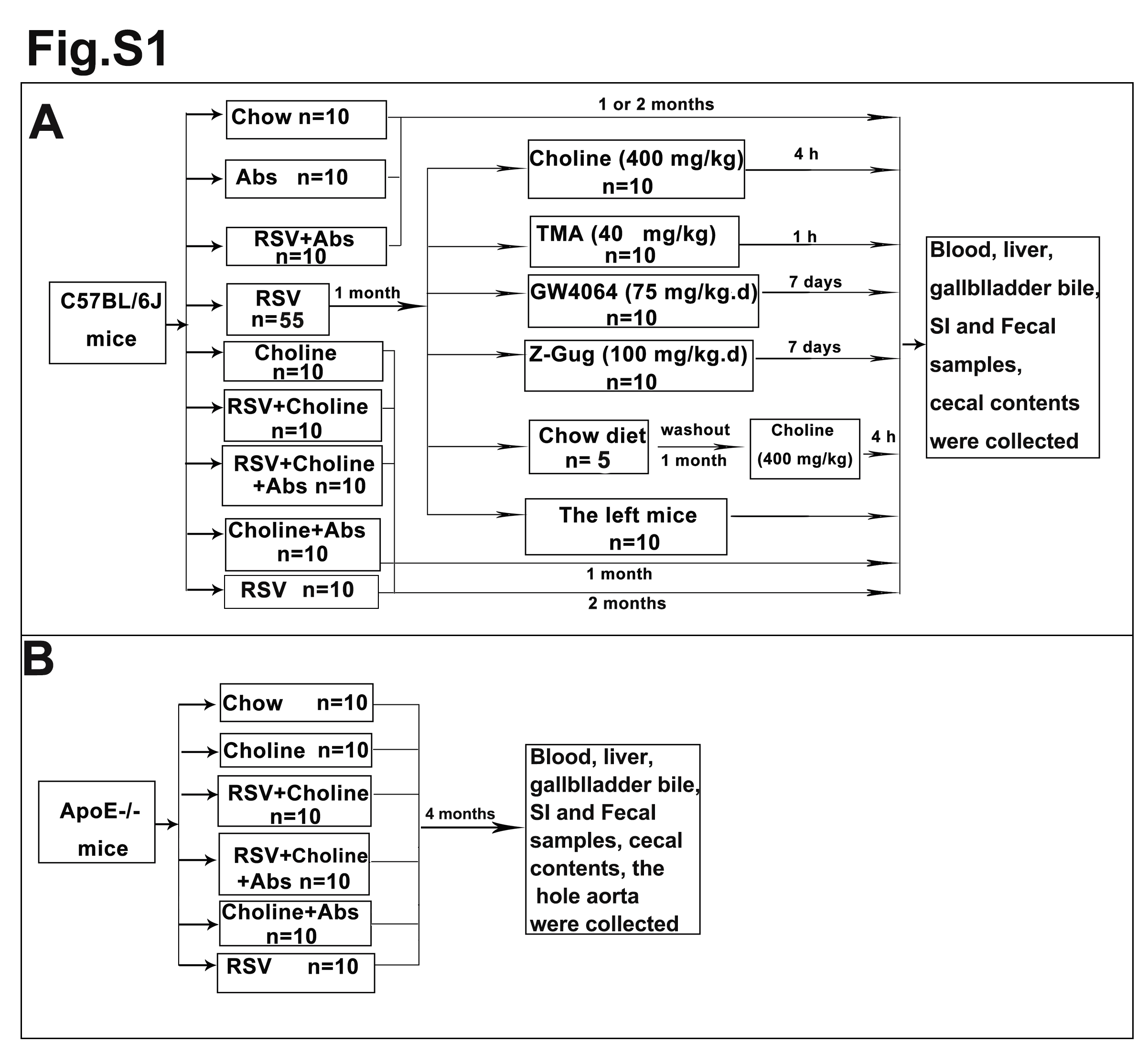

Supplement: Figure S1 — Experimental design. The schematic depicts the overall design of the animal experiment to evaluate the effect of RSV on TMAO-induced AS in both C57BL/6J (A) and ApoE−/− (B) mice. Download [file mbo002162751sf1.tif]

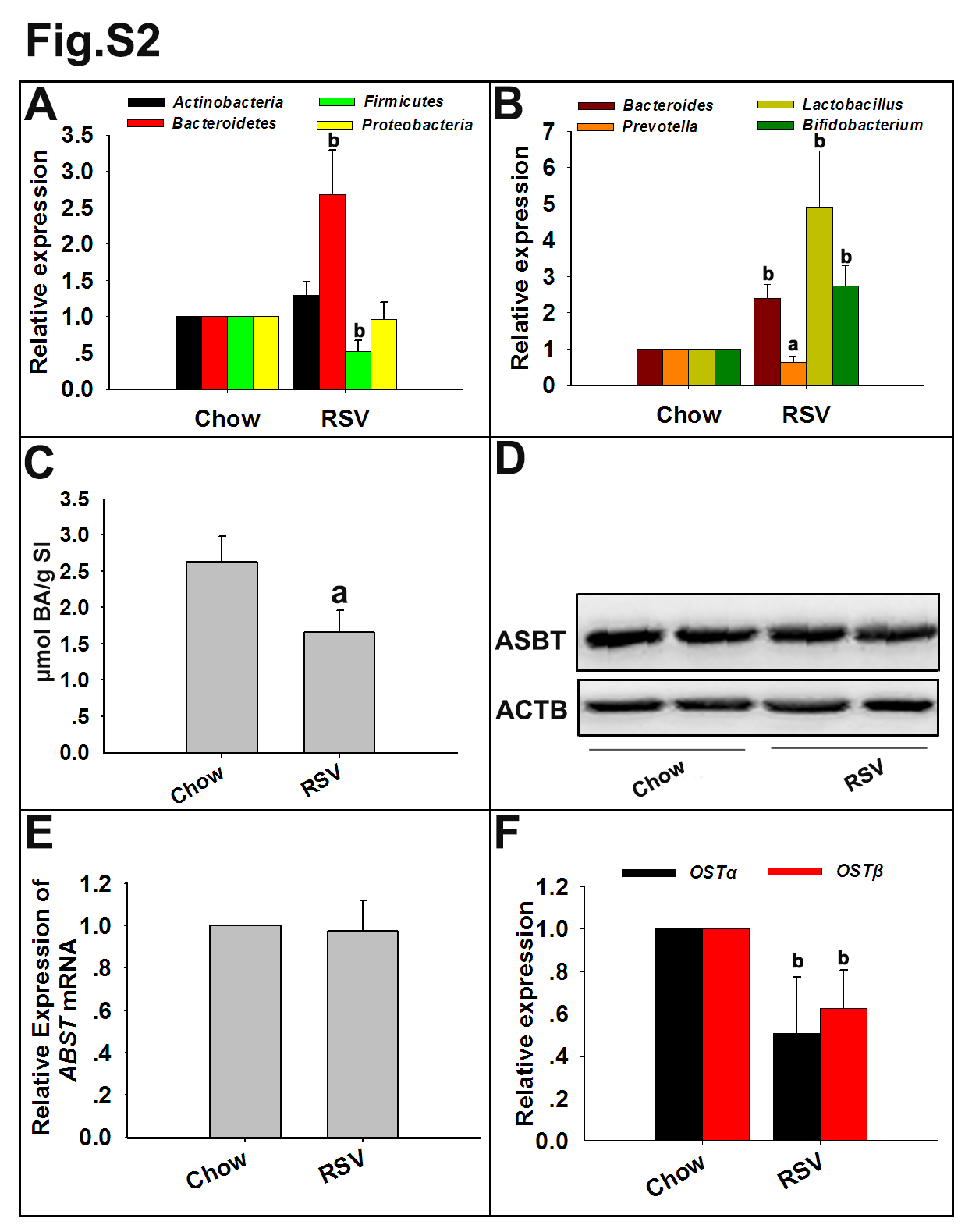

Supplement: Figure S2 — RSV remodeled gut microbiota and decreased ileal BA content without modifying ASBT function. Eight-week-old female C57BL/6J mice (n = 10 per group) were fed a chow diet with or without RSV (0.4%) for 1 or 2 months. (A and B) The relative abundances of the indicated bacterial strains at the phylum level (A) and the genus level (B) in the cecal content were assessed by qPCR assay. (C) Total BA content in ileal tissues. (D) Western blotting was used to detect the expression of ASBT. (E and F) Expression of ASBT gene mRNA (E) and OSTα and OSTβ gene mRNA (F) was determined by qPCR assays. Values are expressed as means ± SD (n = 10). a, P < 0.05; b, P < 0.01 (versus vehicle-treated control group). Download [file mbo002162751sf2.tif]

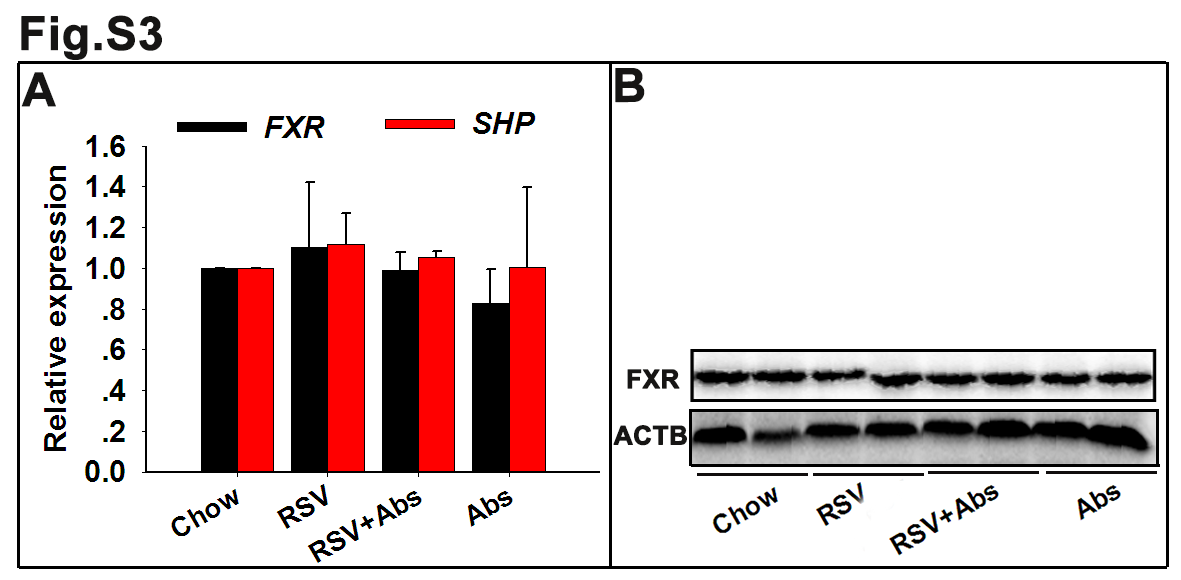

Supplement: Figure S3 — RSV had no effect on liver FXR and SHP gene mRNA expression. Eight-week-old female C57BL/6J mice (n = 10 per group) were fed a chow diet with or without RSV (0.4%) in the presence or absence of Abs for 30 days. Liver tissues were collected. (A) Relative expression levels of the indicated mRNAs in the liver were determined by qPCR assays. (B) FXR expression was analyzed by Western blotting. Values are expressed as means ± SD (n = 10). Download [file mbo002162751sf3.tif]

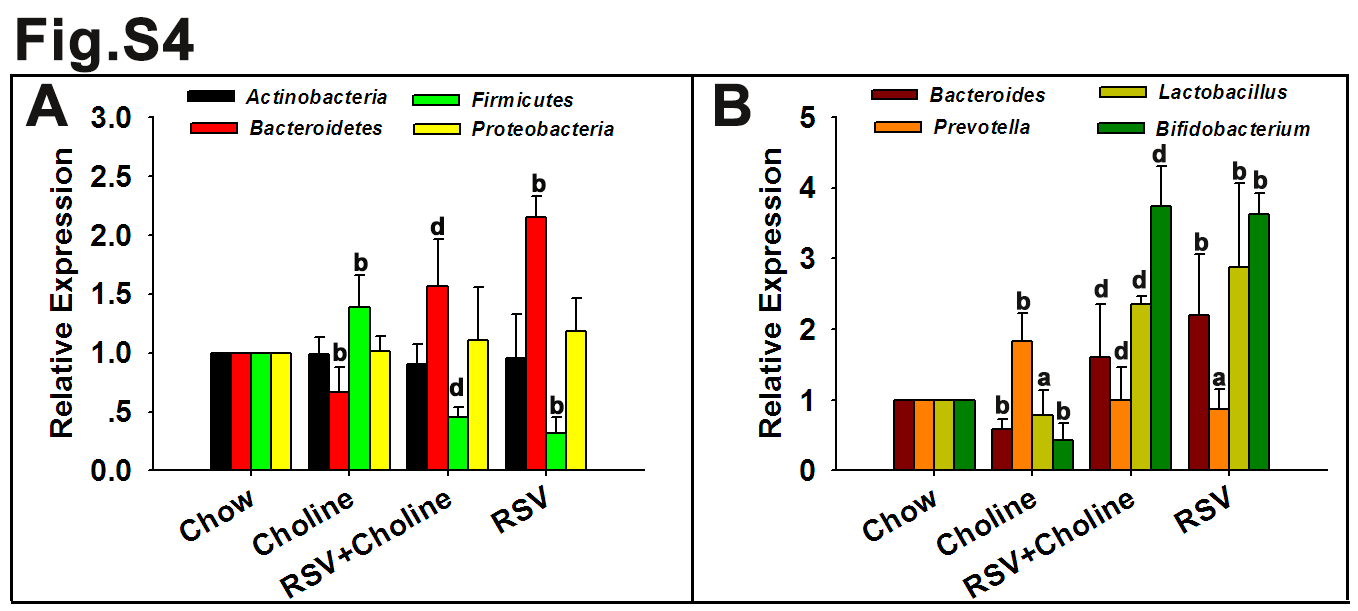

Supplement: Figure S4 — RSV remodeled gut microbiota in choline-fed ApoE−/− mice. Eight-week-old female ApoE−/− mice (n = 10 per group) were fed chow, chow with RSV (0.4%), chow with choline (1%), or chow with choline (1%) plus RSV (0.4%) for 4 months. The relative abundances of the indicated bacterial strains at the phylum level (A) and the genus level (B) in the cecal content were assessed by qPCR assay. a, P < 0.05; b, P < 0.01 (versus vehicle-treated control group); d, P < 0.01 (versus choline-treated group). Download [file mbo002162751sf4.tif]

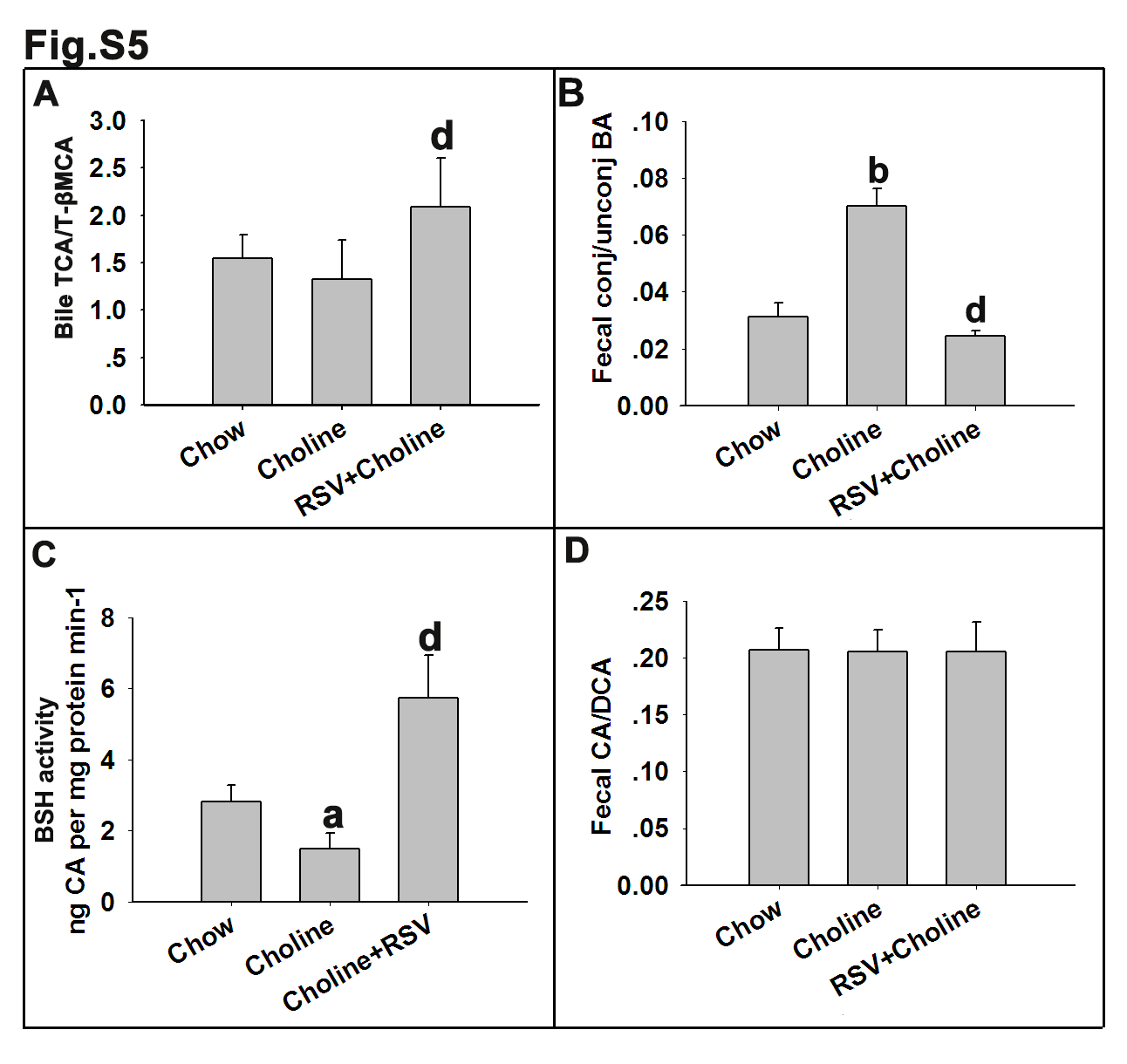

Supplement: Figure S5 — RSV altered the BA composition in gallbladder bile and feces and increased fecal BSH activity in choline-treated ApoE−/− mice. Eight-week-old female ApoE−/− mice (n = 10 per group) were fed chow, chow with choline (1%), or chow with choline (1%) plus RSV (0.4%) for 4 months. The BA composition of gallbladder bile and feces was analyzed by LC/MS. (A) TCA/TβMCA ratios in gallbladder bile. (B) Conjugated/unconjugated BA ratios in fecal samples. (C) Fecal BSH activity. (D) CA/DCA ratios in fecal samples. Values are expressed as means ± SD (n = 10). a, P < 0.05; b, P < 0.01 (versus vehicle-treated control group); d, P < 0.05 (versus choline-treated group). Download [file mbo002162751sf5.tif]
