# Supplementary material for: Resveratrol Attenuates Trimethylamine-N-Oxide (TMAO)-Induced Atherosclerosis by Regulating TMAO Synthesis and Bile Acid Metabolism via Remodeling of the Gut Microbiota
Source: mBio. 2016 Apr 5;7(2):e02210-15. doi: 10.1128/mBio.02210-15 (PMC4817264; doi:10.1128/mBio.02210-15)
Supplement: Table S1 — Primers used for qPCR. [file mbo002162751st1.docx]

**Table. S1 Primers used for qPCR**

| **Name** | **Sequence (5’-3’)** |
| --- | --- |
| *FXR* | Sense: TGAGAACCCACAGCATTTCG  Antisense: GCGTGGTGATGGTTGAATGTC |
| *SHP* | Sense: CGATCCTCTTCAACCCAGATG  Antisense: AGGGCTCCAAGACTTCACACA |
| *FGF15* | Sense: ACGTCCTTGATGGCAATCG  Antisense: GAGGACCAAAACGAACGAAAT T |
| *FMO3* | Sense: GGAACCAGGAATATGGAAG  Antisense: GGTGACCTTCTGAGCTACAT |
| *OSTα* | Sense: TGTTCCAGGTGCTTGTCATCC  Antisense: CCACTGTTAGCCAAGATGGAGAA |
| *OSTβ* | Sense: GATGCGGCTCCTTGGAATTA  Antisense: GGAGGAACATGCTTGTCATGAC |
| *ASBT* | Sense: ACCACTTGCTCCACACTGCTT  Antisense: CGTTCCTGAGTCAACCCACAT |
| *CYP7A1* | Sense:AGCAACTAAACAACCTGCCAGTACTA  Antisense: GTCCGGATATTCAAGGATGCA |
| *GAPDH* | Sense: GCAAAGTGGAGATTGTTGCCAT  Antisense: CCTTGACTGTGCCGTTGAATTT |
| *Firmicutes* | Sense: ATGTGGTTTAATTCGAAGCA  Antisense: AGCTGACGACAACCATGCAC |
| *Proteobacteria* | Sense: CATGACGTTACCCGCAGAAGAAG Antisense: CTCTACGAGACTCAAGCTTGC |
| *Actinobacteria* | Sense: CGCGGCCTATCAGCTTGTTG  Antisense: CCGTACTCCCCAGGCGGGG |
| *Bacteroidetes* | Sense: GGARCATGTGGTTTAATTCGATGAT Antisense: AGCTGACGACAACCATGCAG |
| *Bacteroides* | Sense: GGTGTCGGCTTAAGTGCCAT  Antisense: CGGAYGTAAGGGCCGTGC |
| *Prevotella* | Sense: GGTTCTGAGAGGAAGGTCCCC  Antisense: TCCTGCACGCTACTTGGCTG |
| *Lactobacillus* | Sense: AGCAGTAGGGAATCTTCCA  Antisense: CACCGCTACACATGGAG |
| *Bifidobacterium* | Sense:CTCCTGGAAACGGGTGG Antisense:GGTGTTCTTCCCGATATCTACA |
