# Supplementary material for: Resveratrol Attenuates Trimethylamine-N-Oxide (TMAO)-Induced Atherosclerosis by Regulating TMAO Synthesis and Bile Acid Metabolism via Remodeling of the Gut Microbiota
Source: mBio. 2016 Apr 5;7(2):e02210-15. doi: 10.1128/mBio.02210-15 (PMC4817264; doi:10.1128/mBio.02210-15)
Supplement: Table S2 — Composition of Mega medium. [file mbo002162751st2.docx]

**Table. S2 Composition of Mega Medium**

| **Component** | **Quantity/ L** | **Comments** |
| --- | --- | --- |
| Tryptone Peptone | 10 g |  |
| Yeast Extract | 5 g |  |
| D-glucose | 2 g |  |
| L-Cysteine HCl | 0.5 g |  |
| Potassium Phosphate Buffer | 100 mL | 1 M stock solution, pH 7.2 |
| Vitamin K3 (menadione) | 1 mL | 1 mg/mL in 100% ethanol stock solution |
| MgSO4 .7 H2O | 0.02 g |  |
| NaHCO3 | 0.4 g |  |
| NaCl | 0.08 g |  |
| CaCl2 | 1 mL | 0.8 g/100 mLdH2O stock solution |
| FeSO4.7 H2O | 1 mL | 40 mg/100 mLdH2O stock solution |
| Resazurin | 4 ml | 25 mg resazurin/100 ml of dH20 stock solution |
| Histidine Hematin | 1 ml | 1.2 mg hematin/ml in 0.2 M histidine (pH 8.0) stock solution |
| Tween80 | 2ml | 25% (vol/vol) dH20 stock solution |
| Sodium Acetate | 1 g |  |
| Meat Extract | 5 g |  |
| ATCC Vitamin Mix | 10 ml |  |
| ATCC Trace Mineral Mix | 10 ml |  |
