# Supplementary material for: Resveratrol Attenuates Trimethylamine-N-Oxide (TMAO)-Induced Atherosclerosis by Regulating TMAO Synthesis and Bile Acid Metabolism via Remodeling of the Gut Microbiota
Source: mBio. 2016 Apr 5;7(2):e02210-15. doi: 10.1128/mBio.02210-15 (PMC4817264; doi:10.1128/mBio.02210-15)
Supplement: Table S3 — Retention times of BAs. [file mbo002162751st3.docx]

**Table. S3 Retation times of BAs**

| **Bile acid conjugates** | **Molecular weight** | **Retation time (min)** | **Multiple reaction model** |
| --- | --- | --- | --- |
| βMCA | 408.57 | 5.530 | 407.3 407.3 |
| CA | 408.57 | 8.549 | 407.3 343.0 |
| CDCA | 392.57 | 7.148 | 391.4 391.4 |
| DCA | 392.57 | 11.338 | 391.4 345.4 |
| LCA | 376.57 | 13.094 | 375.4 375.4 |
| TβMCA | 537.69 | 3.982 | 514.4 106.7 |
| TCA | 537.68 | 5.510 | 514.3 123.9 |
| TCDCA | 521.69 | 8.603 | 498.3 106.8 |
| TDCA | 521.69 | 9.201 | 498.3 123.9 |
