# Supplementary material for: Resveratrol Attenuates Trimethylamine-N-Oxide (TMAO)-Induced Atherosclerosis by Regulating TMAO Synthesis and Bile Acid Metabolism via Remodeling of the Gut Microbiota
Source: mBio. 2016 Apr 5;7(2):e02210-15. doi: 10.1128/mBio.02210-15 (PMC4817264; doi:10.1128/mBio.02210-15)
Supplement: Table S4 — Biliary, SI and fecal bile acid composition profile in mice (related to data shown in Fig. 3, 4, and 9 and in Fig. S2 and S5). [file mbo002162751st4.docx]

**Table S4. Biliary, SI and fecal bile acid composition profile in mice (related to**

**Fig. 3, Fig. 4, Fig. 9, Fig. S2 and Fig. S5).**

|  | **C57BL/6J mice** | | **ApoE^-/-^ mice** | | |
| --- | --- | --- | --- | --- | --- |
|  | **Chow** | **RSV** | **Chow** | **Choline** | **RSV+ Choline** |
| **Biliary (mM)** |  |  |  |  |  |
| **TCA** | 101.1 ± 17.3 | 243.0 ± 8.0 ** | 78.8 ± 18.0 | 40.8 ± 3.3 ^**^ | 148.9 ± 32.9 ^##^ |
| **TβMCA** | 65.0 ± 9.6 | 91.8 ± 7.5 ^*^ | 55.7 ± 10.4 | 34.1 ± 7.1 ^*^ | 57.8 ± 10.1 ^##^ |
| **TDCA** | 7.2 ± 1.5 | 13.8 ± 8.5 ^*^ | 5.9 ± 1.6 | 1.6 ± 0.7 ^*^ | 8.6 ± 1.9 ^#^ |
| **TCDCA** | 4.7 ± 1.8 | 6.0 ± 1.2 | 3.4 ± 1.9 | 2.1 ± 0.4 | 3.7 ± 0.3 ^#^ |
|  |  |  |  |  |  |
| **SI (μg/g)** |  |  |  |  |  |
| **TCA** | 688.3 ± 22.4 | 206.4 ± 6.6 ^**^ | 417.1 ± 40.9 | 722.2 ± 91.2 ^**^ | 198.8 ± 10.8 ^##^ |
| **TβMCA** | 293.1 ± 12.0 | 248.7 ± 20.0 ^*^ | 219.6 ± 32.1 | 289.4 ± 27.8 ^*^ | 237.5 ± 25.2 ^#^ |
| **DCA** | 24.4 ± 9.6 | 15.3 ± 6.4 ^*^ | 19.3 ± 9.4 | 9.8 ± 3.3 ^*^ | 9.3 ± 1.9 |
| **CDCA** | 16.9 ± 1.9 | 6.8 ± 1.4 ^*^ | 10.3 ± 5.5 | 19.9 ± 4.7 ^*^ | 6.7 ± 1.6 ^#^ |
| **CA** | 680.5 ± 74.8 | 623.8 ± 20.4 ^*^ | 590.6 ± 27.6 | 418.5 ± 39.0^*^ | 412.7 ± 16.7 |
| **βMCA** | 98.5 ± 18.2 | 96.7 ± 15.4 | 109.7 ± 38.4 | 79.0 ± 11.3 ^*^ | 74.0 ± 14.0 |
|  |  |  |  |  |  |
| **Feces (μg/g)** |  |  |  |  |  |
| **TCA** | 351.8 ± 67.0 | 200.8 ± 34.6 ^**^ | 394.4 ± 46.6 | 600.3 ± 62.2 ^**^ | 385.9 ± 42.7 ^##^ |
| **TβMCA** | 172.1 ± 11.4 | 126.5 ± 78.0 ^*^ | 145.6 ± 18.0 | 217.3 ± 73.6 ^*^ | 167.9 ± 26.8 ^#^ |
| **DCA** | 1.0×10^4^ ± 809.8 | 2.3×10^4^ ± 3290.1^**^ | 1.6×10^4^ ± 2053.8 | 9.9×10^3^ ± 906.5 ^**^ | 1.7 ×10^4^ ± 1463.7 ^##^ |
| **CDCA** | 446.7 ± 50.4 | 468.6 ± 33.5 | 396. 0± 97.5 | 377.6 ± 28.1 | 394.2 ± 55.9 |
| **CA** | 2243.1 ± 111.8 | 4990.3 ± 130.1^*^ | 3316.56 ± 323.5 | 2023.5 ± 21.6 ^**^ | 3562.5 ± 326.4 ^##^ |
| **βMCA** | 1687.7 ± 158.6 | 2430.5 ± 118.1^*^ | 1299.7 ± 192.3 | 805.1 ± 78.6 ^**^ | 1362.7 ± 380.3 ^##^ |
| **LCA** | 608.4 ± 70.0 | 1084.65 ± 147.8 ^**^ | 692.4 ± 75.2 | 513.7 ± 30.7 ^*^ | 696.8 ± 28.5 ^#^ |
| **TCDCA** | 134.8 ± 32.0 | 69.5 ± 3.8 ^*^ | 169.6 ± 62.4 | 137.6 ± 38.6 | 149.8 ± 20.2 |

*^*^p*< 0.05, *^**^p* < 0.01 *vs.* vehicle-treated control group; *^#^p*< 0.05, *^##^p*< 0.01 *vs.* choline-treated group.
