# Supplementary material for: Resveratrol Attenuates Trimethylamine-N-Oxide (TMAO)-Induced Atherosclerosis by Regulating TMAO Synthesis and Bile Acid Metabolism via Remodeling of the Gut Microbiota
Source: mBio. 2016 Apr 5;7(2):e02210-15. doi: 10.1128/mBio.02210-15 (PMC4817264; doi:10.1128/mBio.02210-15)
Supplement: Text S1 — Supplemental materials and methods. Download [file mbo002162751s1.docx]

**TextS1. Materials and Methods**

**1.1 Reagents and antibodies.**

Choline (C7017), TMAO (317594), TMA (W324108), GW4064 (G5172), RSV (R5010) were purchased from Sigma-Aldrich (St. Louis, MO, USA). Standards for CA, CDCA, DCA, lithocholic acid (LCA), tauro-cheno-deoxycholic acid (TCDCA) taurodeoxycholic acid (TDCA) and internal standard D5-CA were all purchased from Sigma-Aldrich (St. Louis, MO, USA). TβMCA (C1899-000) and βMCA (C1895-000) were obtained from Steraloids (Newport, RI, USA). D5-TCA (T008852) was obtained from Toronto Research Chemicals (Toronto, Canada). We purchased Z-Gug (sc-204414) and antibodies against FGF15 (sc-398338), and ASBT (sc-27494) from Santa Cruz Biotechnology (Santa Cruz, CA, USA). Antibodies against FMO3 (Ab126711), FXR (Ab126602), and CYP7A1 (Ab65596) were from Abcam (Cambridge, UK).

**1.2 Animals and treatments**

**1.2.1 *Evaluation of peak TMA and TMAO concentrations in serum.***

The 8-week-old female C57BL/6J mice were made to fast overnight and then administered choline (400 mg/kg) or TMA (40 mg/kg) by intragastric gavage. Blood samples were obtained from the tail veins at 0, 1, 2, 4, 6 and 8 h after the administration of choline or TMA. Serum was separated from the blood by centrifugation and stored at −80°C until required.

**1.2.2 *Determination of the effects of RSV on TMAO and BA synthesis in*** **C57BL/6J mice*.***

. ereontrol experiments, eding aWe fed 8-week-old female C57BL/6J mice a chow diet supplemented with either 0.4% RSV, 1% choline, 1% choline plus 0.4% RSV, in the absence or presence of Abs (0.5 g/L vancomycin, 1 g/L neomycin sulfate, 1 g/L metronidazole, 1 g/L ampicillin) for 1 or 2 months  **(Fig. S1A)**. Mice were weighed every week. No traces of RSV were detected in the commercial diet, as determined by the method reported by Juan et al ([1](#_ENREF_1)). After 1 month, 10 mice of 0.4% RSV-single treated group were washout with a chow die for 1 month before killed. And several mice (n = 10 per group) of the control or RSV-single treated group were made to fast overnight and then administered choline (400 mg/kg) or TMA (40 mg/kg) by intragastric gavage; serum samples were taken at 4 or 1 h after the administration of choline or TMA, respectively. Some mice of the chow or RSV treated group were given Z-Gug (100 mg/kg body weight, n = 10) or GW4064 (75 mg/kg body weight, n = 10) 7 days prior to surgical procedures, respectively. The other mice were euthanized without fasting at the end of 1 or 2 months. Blood samples were collected by retro-orbital bleeding; liver and intestine samples, cecal content were collected immediately following killing by CO2 asphyxiation, then snap-frozen in liquid nitrogen, and stored at −80°C until required. We also collected fecal samples to measure fecal BA excretion and enzymatic activity.

***1.2.3 Determination of the effect of RSV on TMAO-induced AS.***

We fed 8-week-old female ApoE^-/-^ mice a chow diet supplemented with either 1% choline, 0.4% RSV or 1% choline plus 0.4% RSV in the absence or presence of Abs (0.5 g/L vancomycin, 1 g/L neomycin sulfate, 1 g/L metronidazole, 1 g/L ampicillin) for 4 months **(Fig. S1B)**. Mice were weighed every week. After 4 months, mice were euthanized, and their blood, tissues, cecal content and fecal samples were collected as described in *2.2*.

**1.3 Quantitation of TMAO and TMA levels**

We added 75 μL of 80% acetonitrile to 25 μL of plasma to precipitate proteins. As internal standards, d9-(trimethyl) TMAO and d9-(trimethyl) TMA were added to plasma samples for their respective native compounds. After 30 min, samples were centrifuged (14,000 × *g*, 4°C, 30 min) and then analyzed by LC/MS as reported previously ([2](#_ENREF_2)). Briefly, LC/MS analysis was carried out using an Agilent 6410 Series Triple Quadrupole mass spectrometer (Agilent Technologies, Wilmington, DE, USA) equipped with an electrospray ionization source. The capillary voltage was set at +4000 V and heated to 350°C. TMAO and TMA were monitored in multiple reaction monitoring mode using characteristic precursor-product ion transitions: m/z 76→58, and m/z 60→44. An Agilent Technologies 1260 HPLC system was equipped with a G1322A vacuum degasser, a G1312B binary pump, a G1316B column oven, and a G1367D autosampler. Chromatographic separation was performed on an XBridge™ HILIC column (150 × 2.1 mm, internal diameter of 3.5 μm; WATERS) protected by a flex capillary XBridge™ HILIC guard column (10 × 2.1 mm, internal diameter of 3.5 μm; WATERS). The mobile phase containing methanol with 0.1% formic acid (phase A) and water with 0.1% formic acid (phase B) was used at a ratio of 32:68 (phase A: phase B) with a flow rate of 0.25 ml/min. Various concentrations of TMAO and TMA standards were added to control plasma to generate calibration curves that allowed us to quantify plasma TMAO and TMA levels.

**1.4 Evaluation of RSV concentration in plasma and cecal content**

RSV concentration in plasma and cecal content was determined by LC/MS as described previously ([3](#_ENREF_3)). Briefly, plasma samples (100 μL per mice) were stabilized with a freshly-prepared 10 mM solution of ascorbic acid (100 μL) and acidified with 0.6 M acetic acid (100 μL). Cecal content (300 mg) was finely ground in 300 μL of physiological saline using a tissue homogenizer (Ultra-Turrax; IKA Labortechnik, Staufen, Germany) for 3 min at room temperature. Acetone (2 mL) was added to the homogenate, then centrifuged (16,000×g, 15 min, 4 ºC), and the supernatant was evaporated under nitrogen gas. The samples were dissolved in 100 μL of methanol and 10 μL aliquots were subjected to LC/MS analysis.

**1.5 Western blot analysis**

Liver and ileum tissues were collected, lysed, and subjected to western blot analysis as described previously ([4](#_ENREF_4)). Briefly, proteins were resolved by 10–15% sodium dodecyl sulfate polyacrylamide gel electrophoresis and transferred to polyvinylidene difluoride membranes. Following incubation with Tris-buffered saline containing 0.1% Tween-20 with 5% skim milk, blots were probed with primary antibodies at dilutions of 1:500 and 1:1,000 overnight at 4°C, followed by horseradish peroxidase-conjugated secondary antibodies ([Thermo Scientific Lab Vision, Fremont, USA;](http://www.thermoscientific.com/ecomm/servlet/search?searchType=0&searchSubType=6&N=4294967135%204294967089&Ne=4294967089&keyWord=rabbit+secondary+antibodies) 31340 and 31455). Protein bands were visualized using the enhanced chemiluminescence system and densitometric analysis was performed using Scion Image-Release Beta 4.02 software.

**1.6 qPCR assays.**

Primer Express software (Applied Biosystems, Foster City, CA, USA) was used to design oligonucleotide primers. PCR assays were performed in 96-well optical reaction plates using an ABI 7500HT thermal cycler (Applied Biosystems) as described previously ([5](#_ENREF_5)). Briefly, total RNA was extracted from liver and ileum tissues using Trizol reagent, and 1 µg of RNA reverse transcribed into cDNA using the primers indicated in **Table. S1.** A 25-µL reaction containing 4 µg of cDNA, 12.5 µL of SYBR Master mix, and 1 µL of each primer was subjected to thermal cycling, involving an initial denaturation step at 95°C for 10 min and 45 cycles of ampliﬁcation (95°C for 15 s, 60°C for 1 min). All ampliﬁcation reactions were performed in triplicate, and the averages of the threshold cycle (Ct) were used to interpolate curves using 7300 System SDS Software. Results are presented as the ratio of each mRNA compared with the expression level of glyceraldehyde 3-phosphate dehydrogenase mRNA.

**1.5 Determination of enzymatic activity of hepatic FMO.**

The enzymatic activity of hepatic FMO was investigated according to previously published methods with minor modiﬁcations ([2](#_ENREF_2)). Briefly, 1 mg liver protein homogenate, 100 μM TMA and 100 mM nicotinamide adeninedinucleotide phosphate were mixed in 250 μl reaction in 10 mM HEPES (pH 7.4). After 8 h, 0.2 N formic acid was used to stop the reaction, followed by ﬁltering through a 3 K cut off spin ﬁlter, and then snap frozen and stored at -80 until time of analysis. For analyses, internal standard was added to the thawed ﬁltrate, which was then injected onto LC/MS to measure the oxidized product TMAO as described above.

**1.7 16S rRNA sequencing of the intestinal microbiome**

Total DNA was isolated and purified from caecum content (*n* = 5 per group) using the PowerSoil DNA Isolation Kit (Mo Bio Laboratories, Inc., Carlsbad, CA, USA). The quality of DNA samples was assessed by gel electrophoresis, and spectrophotometry at 260 and 280 nm. Only DNA samples of sufficient quality were subjected to PCR, with primers targeting the hypervariable V4-V5 regions of 16S RNA. Amplicons were extracted from 2% agarose gels and purified using the AxyPrep DNA Gel Extraction Kit (Axygen Biosciences, Union City, CA, USA) according to the manufacturer’s instructions. Samples were then quantified using a QuantiFluor™-ST fluorometer (Promega, Madison, WI, USA). Purified amplicons were pooled in equimolar quantities and paired-end sequenced (2 × 250) on an Illumina MiSeq platform according to standard protocols. The raw reads were deposited into the NCBI Sequence Read Archive database. Raw fastq files were demultiplexed, quality-filtered using QIIME (version 1.17) with the following criteria: (i) The 250 bp reads were truncated at any site receiving an average quality score <20 over a 10 bp sliding window, discarding the truncated reads that were shorter than 50bp. (ii) exact barcode matching, 2 nucleotide mismatch in primer matching, reads containing ambiguous characters were removed. (iii) only sequences that overlap longer than 10 bp were assembled according to their overlap sequence. Reads which could not be assembled were discarded. Operational Units (OTUs) were clustered with 97% similarity cutoff using UPARSE（version 7.1 <http://drive5.com/uparse/>) and chimeric sequences were identified and removed using UCHIME. The phylogenetic affiliation of each 16S rRNA gene sequence was analyzed by RDP Classifier (http://rdp.cme.msu.edu/) against the silva (SSU115)16S rRNA database using confidence threshold of 70%.([6](#_ENREF_6)) Differences between vehicle- and RSV-treated C57BL/6J mice, or choline- and RSV plus choline-fed ApoE^-/-^ mice were analyzed using non-parametric Student’s *t*-test.

**1.8 Quantitation of TMA production from choline *in vitro.***

The TMA synthesis potential of cecal content was determined *in vitro* as described before ([7](#_ENREF_7), [8](#_ENREF_8)). Briefly, cecal content (50 mg) was mixed with 500 μL phosphate buffered saline (PH 7.2) and centrifuged (200×g, 5 min, 4 ºC). Thereafter, 100 μL of bacterial suspension was inoculated into 1 mL of Mega Medium **(Table. S2)** supplemented with 15 mM choline chloride in a 96-well deep-well plate sealed with sterile foil, and incubated anaerobically for 48 h at 37°C. Cell culture supernatants were harvested by centrifugation at 4°C. Samples were ﬁltered through a 0.2 μm ﬁlter (Millipore) and 10 μL aliquots were subjected to LC/MS analysis.

**1.9 Analysis of microbiota from cecal contents by qPCR assay.**

About 100 mg of cecal content from each animal was taken and DNA was isolated

using the QIAamp DNA Stool Mini Kit (Qiagen, Venlo, Netherlands) according to the manufacturer's instructions. DNA was quantified using Infinite M200 ProNanoQuant (Tecan, Switzerland). Integrity of the DNA samples was evaluated using 0.8% agarose gel. Real-time quantification of *Bacteroidetes*, *Firmicutes, Actinobacteria ,Proteobateria*, *Lactobacillus*, *Bifidobacterium, Bacteroides* and *Prevotella,* was performed as described above. The specific primers used for these groups were indicated in **Table. S1.**

**1.10 BA measurements**

***1.10.1Total BA detection***

At the end of experiments, mice were subjected to fasting for 12 h and then euthanized. Serum, gallbladder bile, liver, whole SI and fecal samples were collected. Intestinal tissues were removed and flushed with ice-cold phosphate-buffered saline. The intestine was cut open longitudinally, mucosa was gently scraped off, and the tissue flash frozen in liquid nitrogen. For fecal BA excretion, mice were individually housed in wire bottom cages and stools were collected over 3 days and then air-dried, weighted and grounded. The BA in each sample was extracted and determined with Total BAs Test Wako (Wako Pure Chemical Industries Ltd., Osaka, Japan) as previously described ([Yu et al., 2000](#_ENREF_10).([9](#_ENREF_9)) The BA pool size was determined as the total BA content of gallbladder bile, liver, and the SI lumen.

***1.10.2 LC/MS analysis***

SI tissues, fecal and gallbladder bile samples were prepared as described before ([10](#_ENREF_10)). Briefly, SI and fecal samples were prepared by mixing 40 mg of SI tissues or feces in 400 μL of 80% acetonitrile, respectively. We added 100 µg/mL D5-TCA and D5-CA as an internal standard to achieve a final concentration of 0.2 µg/mL. After 30 min, samples were centrifuged (14,000 × *g*, 30 min) and 5 μL aliquots were collected for LC/MS analysis. Gallbladder bile samples were diluted 1:400 with 80% acetonitrile, 100 µg/mL D5-TCA and D5-CA were added to a final concentration of 0.2 µg/mL. After 15 min, samples were centrifuged (14,000 × *g*, 30 min) and then analyzed by LC/MS. Bile acids were analyzed on an Acquity UPLC system coupled to a Waters Xevo TQ-S MS (Waters, Manchester, UK) with an Acquity HSS T3 (2.1× 100 mm, 1.7 μm) column (Waters) and gradient elution with 10 mM formic acid in water and 10 mM formic acid in acetonitrile:methanol (35:65) as mobile phases. Cone voltage was 70 V and collision energy 2 eV for unconjugated bile acids and 90 V and 65 eV for taurine conjugates. For metabolite quantification, calibration curves (0.001–10 µg/mL) were prepared in 80% acetonitrile with D5-CA (0.2 µg/mL) or D5-TCA (0.2 µg/mL). The equations for standard curves were calculated using weighted (1/y) linear regression of internal ratios (analyte/internal standard peak area) versus analyte concentrations. Linearity was assessed and accepted as the range of concentrations in which the correlation coefficient (R^2^) was ≥ 0.995, with a precision and accuracy of ≤ 20%. The limit of detection was assessed as the lowest concentration where the signal intensity was at least three times greater than the background level. The internal standard working solution (0.2 µg/mL) was prepared by dilution of a stock solution with 80% acetonitrile. All standard solutions were stored at −20°C. Retention times of analytes are presented in **Table S3**.

**1.11 BSH activity**

According to the method of Flores ([11](#_ENREF_11)), fecal proteins were prepared from 0.5 g of fecal samples in 5 mL of PBS (pH 7.4) using sonication. Bacterial BSH activity was measured based on the generation of CA from TCA in the feces. Brieﬂy, incubation was carried out in 3 mM sodium acetate buffer (pH 5.2) containing 0.1 mg/mL fecal protein and 100 µg/mL D5-TCA in a volume of 200 µL. After a 20 min incubation at 37°C, reactions were stopped by plunging the samples into dry ice. We added 100 µL of methanol to the reaction mix and centrifuged samples (14,000 × *g*, 20 min), then analyzed 5 µL of the supernatant by LC/MS.

**1.12 Quantitation of AS**

Atherosclerotic lesions were quantified by en face analysis of the aorta (including aortic arch, thoracic and abdominal regions) and by cross-sectional analysis of the aortic root, along with the detection of TC contents in the aortas as previously reported ([12](#_ENREF_12), [13](#_ENREF_13)). For en face preparations, the aorta was opened longitudinally and stained with Oil-red O (Sigma-Aldrich, St. Louis, MO, USA) to detect lipids and to determine the lesion area. En face images of the aorta were taken with a Canon EOS 7D digital camera (Canon, Tokyo, Japan) and analyzed using ImagePro Plus. Atherosclerotic lesions of the aorta were expressed as a percentage of the total surface area. To measure the atherosclerotic lesions at the aortic sinus, the upper sections of hearts were embedded in OCT compound (Sigma-Aldrich, St. Louis, MO, USA) and frozen at −20°C. Sections (10-µm thickness) were collected, beginning at the aortic root and extending for 400 µm, as described before ([14](#_ENREF_14)). Lesions from 10 alternating sections were stained with Oil-Red-O and haematoxylin, then were quantified using Optimas Image Analysis software (Bioscan Inc., Edmonds, WA, USA). The TC contents of the mouse aortas were determined by enzymatic assays.

**1.13 Detection of aortic root lesions by ultrasound**

A high frequency ultrasound system (Vevo 2100, Visualsonics, Toronto, Canada) equipped with a linear array transducer (MS 550D, 22–55 MHz) was used to detect the atherosclerotic lesions at the aortic sinus as described previously ([15](#_ENREF_15)). Briefly, ApoE^-/-^ mice were anesthetized with an intraperitoneal injection of 50 mg/kg pentobarbital sodium (1% in normal saline). Mice were then placed on a heated procedural board and limbs were taped to ECG electrodes coated with electrode cream; a rectal thermometer was inserted to assist with maintaining normothermia (37°C internal temperature). The fur at the imaging location was shaved and warm ultrasound gel was liberally applied to ensure optimal image quality. The aortic sinus was imaged and visualized using a long-axis view; a CINE loop of 100 frames was stored for later off-line analysis. The time gain compensation curve was adjusted to produce a uniform intensity of echoes. The gain was set to 30 dB and the dynamic range to 65 dB. To reduce variability, image parameters remained constant throughout the experiment. All examinations were performed by an experienced operator, with all measurements repeated three times at the same site. A total of 180 aortic sinus regions of interest from 60 ApoE^-/-^ mice (n=10 per group), were analyzed.

**1.14 Detection of liver cholesterol, serum cholesterol contents**

Blood and liver samples from ApoE^-/-^ mice were collected at the end of treatment. Collected serum was aliquoted and frozen at −80°C until required. Liver cholesterol content and serum TC levels were measured enzymatically, in triplicate, using commercially available kits (Nanjing Jiancheng Bioengineering Institute, Nanjing, China).

**References**

1. **Juan ME, Lamuela-Raventos RM, de la Torre-Boronat MC, Planas JM.** 1999. Determination of trans-resveratrol in plasma by HPLC. Anal Chem **71:**747-750.

2. **Bennett BJ, de Aguiar Vallim TQ, Wang Z, Shih DM, Meng Y, Gregory J, Allayee H, Lee R, Graham M, Crooke R, Edwards PA, Hazen SL, Lusis AJ.** 2013. Trimethylamine-N-oxide, a metabolite associated with atherosclerosis, exhibits complex genetic and dietary regulation. Cell Metab **17:**49-60.

3. **Chen ML, Yi L, Jin X, Xie Q, Zhang T, Zhou X, Chang H, Fu YJ, Zhu JD, Zhang QY, Mi MT.** 2013. Absorption of resveratrol by vascular endothelial cells through passive diffusion and an SGLT1-mediated pathway. Journal of Nutritional Biochemistry **24:**1823-1829.

4. **Chen ML, Yi L, Jin X, Liang XY, Zhou Y, Zhang T, Xie Q, Zhou X, Chang H, Fu YJ, Zhu JD, Zhang QY, Mi MT.** 2013. Resveratrol attenuates vascular endothelial inflammation by inducing autophagy through the cAMP signaling pathway. Autophagy **9:**2033-2045.

5. **Chen CY, Yi L, Jin X, Zhang T, Fu YJ, Zhu JD, Mi MT, Zhang QY, Ling WH, Yu B.** 2011. Inhibitory Effect of Delphinidin on Monocyte-Endothelial Cell Adhesion Induced by Oxidized Low-Density Lipoprotein via ROS/p38MAPK/NF-kappa B Pathway. Cell Biochemistry And Biophysics **61:**337-348.

6. **Amato KR, Yeoman CJ, Kent A, Righini N, Carbonero F, Estrada A, Gaskins HR, Stumpf RM, Yildirim S, Torralba M, Gillis M, Wilson BA, Nelson KE, White BA, Leigh SR.** 2013. Habitat degradation impacts black howler monkey (Alouatta pigra) gastrointestinal microbiomes. ISME J **7:**1344-1353.

7. **Craciun S, Balskus EP.** 2012. Microbial conversion of choline to trimethylamine requires a glycyl radical enzyme. Proceedings of the National Academy of Sciences of the United States of America **109:**21307-21312.

8. **Romano KA, Vivas EI, Amador-Noguez D, Rey FE.** 2015. Intestinal Microbiota Composition Modulates Choline Bioavailability from Diet and Accumulation of the Proatherogenic Metabolite Trimethylamine-N-Oxide. Mbio **6**.

9. **Yu C, Wang F, Kan M, Jin C, Jones RB, Weinstein M, Deng CX, McKeehan WL.** 2000. Elevated cholesterol metabolism and bile acid synthesis in mice lacking membrane tyrosine kinase receptor FGFR4. J Biol Chem **275:**15482-15489.

10. **Sayin SI, Wahlstrom A, Felin J, Jantti S, Marschall HU, Bamberg K, Angelin B, Hyotylainen T, Oresic M, Backhed F.** 2013. Gut microbiota regulates bile acid metabolism by reducing the levels of tauro-beta-muricholic acid, a naturally occurring FXR antagonist. Cell Metab **17:**225-235.

11. **Flores R, Shi J, Gail MH, Ravel J, Goedert JJ.** 2012. Assessment of the human faecal microbiota: I. Measurement and reproducibility of selected enzymatic activities. Eur J Clin Invest **42:**848-854.

12. **Wang D, Xia M, Yan X, Li D, Wang L, Xu Y, Jin T, Ling W.** 2012. Gut microbiota metabolism of anthocyanin promotes reverse cholesterol transport in mice via repressing miRNA-10b. Circ Res **111:**967-981.

13. **Peng N, Meng N, Wang SQ, Zhao F, Zhao J, Su L, Zhang SL, Zhang Y, Zhao BX, Miao JY.** 2014. An activator of mTOR inhibits oxLDL-induced autophagy and apoptosis in vascular endothelial cells and restricts atherosclerosis in apolipoprotein E-/- mice. Scientific Reports **4**.

14. **Paigen B, Morrow A, Holmes PA, Mitchell D, Williams RA.** 1987. Quantitative assessment of atherosclerotic lesions in mice. Atherosclerosis **68:**231-240.

15. **Niu L, Qian M, Yang W, Meng L, Xiao Y, Wong KK, Abbott D, Liu X, Zheng H.** 2013. Surface roughness detection of arteries via texture analysis of ultrasound images for early diagnosis of atherosclerosis. PLoS One **8:**e76880.
